# *Helicobacter pylori*-Induced HB-EGF Upregulates Gastrin Expression via the EGF Receptor, C-Raf, Mek1, and Erk2 in the MAPK Pathway

**DOI:** 10.3389/fcimb.2017.00541

**Published:** 2018-01-15

**Authors:** Niluka Gunawardhana, Sungil Jang, Yun Hui Choi, Youngmin A. Hong, Yeong-Eui Jeon, Aeryun Kim, Hanfu Su, Ji-Hye Kim, Yun-Jung Yoo, D. Scott Merrell, Jinmoon Kim, Jeong-Heon Cha

**Affiliations:** ^1^Department of Oral Biology, Oral Science Research Center, Department of Applied Life Science, The Graduate School, BK21 Plus Project, Yonsei University College of Dentistry, Seoul, South Korea; ^2^Department of Basic Sciences, Faculty of Dental Sciences, University of Peradeniya, Peradeniya, Sri Lanka; ^3^Microbiology and Molecular Biology, Key Laboratory of Oral Medicine, Guangzhou Institute of Oral Disease, Stomatology Hospital of Guangzhou Medical University, Guangzhou, China; ^4^Department of Dental Hygiene, Jeonju Kijeon College, Jeonju, South Korea; ^5^Department of Microbiology and Immunology, Uniformed Services University of the Health Sciences, Bethesda, MD, United States

**Keywords:** *Helicobacter pylori*, gastrin, gastric cancer, HB-EGF, Erk

## Abstract

*Helicobacter pylori* is associated with hypergastrinemia, which has been linked to the development of gastric diseases. Although the molecular mechanism is not fully understood, *H. pylori* is known to modulate the Erk pathway for induction of gastrin expression. Herein we found that an epidermal growth factor (EGF) receptor kinase inhibitor significantly blocked *H. pylori*-induced gastrin promoter activity, suggesting involvement of EGF receptor ligands. Indeed, *H. pylori* induced mRNA expression of EGF family members such as amphiregulin, EGF, heparin-binding EGF-like growth factor (HB-EGF), and transforming growth factor-α. Of these, specific siRNA targeting of HB-EGF significantly blocked *H. pylori*-induced gastrin expression. Moreover, *H. pylori* induced HB-EGF ectodomain shedding, which we found to be a critical process for *H. pylori*-induced gastrin expression. Thus, we demonstrate a novel role for human mature HB-EGF in stimulating gastrin promoter activity during *H. pylori* infection. Further investigation using specific siRNAs targeting each isoform of Raf, Mek, and Erk elucidated that the mechanism underlying *H. pylori*-induced gastrin expression can be delineated as the sequential activation of HB-EGF, the EGF receptor, C-Raf, Mek1, and the Erk2 molecules in the MAPK pathway. Surprisingly, whereas Erk2 acts as a potent activator of gastrin expression, siRNA knockdown of Erk1 induced gastrin promoter activity, suggesting that Erk1 typically acts as a repressor of gastrin expression. Elucidation of the mechanism of gastrin modulation by HB-EGF-mediated EGF receptor transactivation should facilitate the development of therapeutic strategies against *H. pylori*-related hypergastrinemia and consequently gastric disease development, including gastric cancers.

## Introduction

*Helicobacter pylori* colonizes the gastric mucosa of over half of the world's population (Polk and Peek, 2010) and causes chronic gastritis, peptic ulcers, gastric adenocarcinoma and mucosa-associated lymphoid tissue (MALT) lymphoma (Ernst and Gold, 2000; Peek and Crabtree, 2006). *H. pylori* infection is associated with an increased risk of development of gastric cancer, which is currently the third leading cause of cancer-related deaths worldwide (Ferlay et al., 2014). Accordingly, the international agency for research on cancer (IARC) classified *H. pylori* as group 1 carcinogen in 1994 (IARC Working Group on the Evaluation of Carcinogenic Risks to Humans, 1994).

The genome of most *H. pylori* strains carries the *cag* pathogenicity island (*cag*PAI), which consists of about 32 genes including *cagA*. The *cag*PAI encodes a functional type IV secretion system (T4SS) that injects the effector protein CagA into host cells. Within the cells, CagA is phosphorylated by host cell kinases, forms a complex with Src homology region 2-containing phosphatase 2 (SHP-2) (Higashi et al., 2002b), and alters multiple host signaling pathways (Higashi et al., 2002a,b, 2004; Neel et al., 2003; Tsutsumi et al., 2006). Additionally, interaction of the T4SS itself with host cells also modulates intracellular signaling pathways such as the mitogen-activated protein kinase (MAPK) pathway. Furthermore, CagL, which is located at the surface of the T4SS apparatus, interacts with integrin on the gastric epithelial cells (Kwok et al., 2007) and triggers integrin-mediated signaling.

Of the hormones produced in the stomach, gastrin plays a key role in gastric physiology. Gastrin is mainly secreted by the G cells in the antro-pyloric mucosa of the stomach and functions to stimulate gastric acid secretion by the parietal cells. Besides stimulation of gastric acid secretion, gastrin is also known to be involved in various cellular processes such as proliferation, apoptosis, angiogenesis, motility, and increased invasion (Dockray, 1999; Peach and Barnett, 2000; Wroblewski et al., 2002).

*H. pylori*-infected patients show elevated levels of serum gastrin (Levi et al., 1989; Prewett et al., 1991) and eradication of *H. pylori* causes a reduction in the gastrin concentration (Park et al., 1993). Using rodent models, the relationship between hypergastrinemia and *H. pylori* infection has been demonstrated (Lichtenberger et al., 1995). For example, transgenic mice expressing gastrin under the transcriptional control of the insulin promoter developed gastric malignancies in 18–20 months without *H. pylori* infection. Moreover, *H. pylori* infection accelerated the development of gastric cancer such that cancers occurred by 6 months (Wang et al., 1993; Fox et al., 2003).

The epidermal growth factor (EGF) family includes amphiregulin (AR), EGF, heparin-binding EGF-like growth factor (HB-EGF), transforming growth factor (TGF)-α, beta-cellulin (BTC), epigen, and epiregulin. Previous analysis of the amino acid sequence of the HB-EGF precursor (proHB-EGF) revealed a signal sequence (residues 1–23), an extracellular domain (residues 24–159), a transmembrane domain (residues 160–184), and a carboxyl terminal cytoplasmic domain (residues 185–208; Naglich et al., 1992). On the cell surface, proHB-EGF is cleaved and released as mature/soluble HB-EGF (residues 63–148) (Higashiyama et al., 1991). The released HB-EGF is able to act as a mitogen by binding to the EGF receptor in an autocrine, paracrine, or endocrine manner (Riese and Stern, 1998), while the remaining cell surface-bound proHB-EGF is able to function as a juxtacrine growth factor (Singh et al., 2007).

MAPK pathways are chiefly involved in the regulation of fundamental cellular processes such as growth, proliferation, differentiation, migration, and apoptosis (Dhillon et al., 2007). The Raf-Mek-Erk pathway represents the best studied MAPK pathway, and is generally regulated by the activation of cell surface receptors that are called receptor tyrosine kinases (RTK). Once the RTK has been stimulated by ligand binding, activation of a series of adapter proteins leads to conversion of inactive Ras into GTP bound active Ras. Activated Ras then phosphorylates its downstream substrate, Raf (Katz et al., 2007). In humans, the Raf kinase family is composed of 3 members, known as A-Raf, B-Raf, and C-Raf (Raf-1) (Roskoski, 2010). The Raf proteins have restricted substrate specificity and phosphorylate their downstream kinase molecules, Mek1 and Mek2. Activated Mek1 and Mek2 then phosphorylate their only known substrates, Erk1 and Erk2. The activated Erks can then function as homodimers or heterodimers to phosphorylate many fundamental cytoplasmic targets as well as nuclear targets: kinases, phosphatases, transcription factors and cytoskeletal proteins (Casar et al., 2008).

The MAPK signaling pathways are involved in *H. pylori*-mediated cellular functions, as well as gene expression (Keates et al., 1999; Meyer-ter-Vehn et al., 2000; Zhu et al., 2005; Chen et al., 2006; Ding et al., 2008). Furthermore, several studies have shown that the Erk pathway is important in *H. pylori*-induced, as well as EGF-induced, gastrin expression (Chupreta et al., 2000; Zhou et al., 2011; Wiedemann et al., 2012). However, the molecular mechanisms and signaling cascades involved in the pathway are not completely understood. Herein we describe studies designed to explore the molecular mechanism by which the HB-EGF-mediated Erk pathway contributes to *H. pylori*-induced gastrin expression.

## Materials and methods

### *H. pylori* strains and culture

G27 (Xiang et al., 1995), G27Δ*cagA*, G27Δ*cagL*, G27ΔPAI, K74 (Jones et al., 2009), K74Δ*cagA*, K74Δ*cagL*, and PMSS1 (Arnold et al., 2011) *H. pylori* strains were used in this study. *H. pylori* strains G27 and PMSS1 were originally isolated from an endoscopy patient from Grosseto Hospital, Tuscany in 1994 (Italy) (Covacci et al., 1993; Xiang et al., 1995) and a gastroenterology clinic, Sydney in 1997 (Australia) (Arnold et al., 2011), respectively, and have been used extensively in *H. pylori* research. G27 and PMSS1 are part of the Merrell lab collection and were originally obtained from the laboratories of Stanley Falkow (G27) and Anne Müller and Manuel Amieva (PMSS1).

All *H. pylori* strains were cultured on Columbia blood agar base (BD, Franklin Lakes, NJ, USA) supplemented with 5% defibrinated horse blood (Hanil Komed), 10 μg/ml vancomycin (Sigma-Aldrich), 2.5 U/ml polymyxin B (Sigma-Aldrich), 5 μg/ml trimethoprim (Sigma-Aldrich), 8 μg/ml amphotericin B (Amresco), and β-cyclodextrin (Sigma-Aldrich), at 37°C in a microaerobic atmosphere, as previously described (Jones et al., 2009). Microaerobic conditions were generated using a CampyGen sachet (Oxoid) in a gas pack jar. Liquid cultures of *H. pylori* were grown in Brucella broth (BD) containing 10% fetal bovine serum (FBS, Gibco) and 10 μg/ml vancomycin, with shaking at 110 rpm in a microaerobic condition at 37°C.

### Ethics statement

Korean K74 *H. pylori* clinical isolate is part of the Cha lab collection, and previously written informed consent was received from each patient, and the protocol was approved by the Institutional Review Board of Human Research at the Catholic University of Korea (Jones et al., 2009). K74 was anonymized upon acquisition.

### Construction of *H. pylori* isogenic mutant strains in G27 and K74

The G27 and K74 Δ*cagA* mutant strains were generated as described previously (Amieva et al., 2002). Briefly, chloramphenicol acetyltransferase (*cat*) gene (Wang and Taylor, 1990) was amplified and inserted at position 1053 of the G27 *cagA* gene. The resulting G27 *cagA*::*cat* fragment was ligated into pGEM-T Easy vector system (Promega), yielding pDSM729. Plasmid pDSM729 was then transformed into strain G27 and K74 by natural transformation, to yield G27Δ*cagA* and K74Δ*cagA*. The G27ΔPAI mutant was also constructed as described elsewhere (Galgani et al., 2004). Briefly, genomic DNA of strain G27-MA ΔPAI (Jones et al., 2009), which contains a kanamycin resistance gene (*aphA-3* from *Campylobacter coli*) cloned between regions of DNA that correspond to the upstream and downstream regions flanking the *cag* PAI region, was amplified with primers Del-PAI-F: 5′-CCA AAT TTT ATA GGA TTC GCG CTC-3′ and GRace1: 5′-GGT TGC ACG CAT TTT CCC-3′. The resulting ~3 kb fragment was ligated into pGEM-T Easy vector system, yielding pDSM730. Plasmid pDSM730 was then transformed into G27 by natural transformation, to yield G27ΔPAI. *H. pylori* transformants were selected on the Columbia blood agar plates supplemented with 25 μg/ml chloramphenicol (Amresco) or 25 μg/ml kanamycin (Sigma-Aldrich). Mutations were confirmed by both PCR and DNA sequencing analysis (Cosmo Genetech).

The G27 and K74 Δ*cagL* mutant strains were constructed as follows. The flanking regions surrounding *cagL* were PCR amplified using *Pfu* polymerase (Intron Biotechnology) with G27 or K74 genomic DNA as template and a series of PCR reactions as follows: G27, upstream flanking region was amplified with primer G27 *cagL* outside F: 5′-AAG TGG CTA TGC AAA AAG CGA CCC-3′ and primer *cagL* XhoI SmaI F: 5′-TGT CAA CTC GAG CCT CCC GGG ATG ATT TTT CTG AGA CGA CAA G-3′. The G27 downstream region was amplified with primer *cagL* XhoI SmaI R: 5′-AAT CAT CCC GGG AGG CTC GAG TTG ACA ATA ACT TTA GAG CTA GC-3′ and primer *cagL* outside R: 5′-GTG CCT GAT GAG TGG AGA ACG CC-3′. The flanking regions of K74 were amplified in a similar manner using upstream primers (K74 *cagL* outside F: 5′-AAG TGG CTA TGC AAA AAG CGA CCC-3′ and *cagL* XhoI SmaI F-K74: 5′-TAG CTC TAA AGT TAT TGT CAA CTC GAG CCT CCC GGG ATA ATT TTT CTG AGA CAA CAA G-3′) and downstream primers (*cagL* XhoI SmaI R-K74: 5′-TTG TTG TCT CAG AAA AAT TAT CCC GGG AGG CTC GAG TTG ACA ATA ACT TTA GAG CTAG-3′ and *cagL* outside R-7.13: 5′-GAG TGG AGA ACA CCT GAA ATT G-3′).

The G27 and K74 *cagL* XhoI SmaI F and R primers were designed to contain overlapping sequences as well as XhoI and SmaI restriction enzyme sites. Thus, the 5′ and 3′ regions were next fused together by splicing-by-overlap-extension (SOE) PCR (Horton et al., 1990, 1993), and the fused PCR products were cloned into the pGEM-T Easy vector. The *kan-sacB* cassette was removed by XhoI and SmaI restriction enzyme digest from the pKSF-II plasmid (Copass et al., 1997) and cloned into the SOE fusion constructs digested with the same enzymes. The proper insertion of the *kan-sacB* fragment between the *cagL* flanking segments was verified by enzyme digestion and DNA sequencing analysis. The pG27 and pK74 Δ*cagL*::*kan-sacB* constructs were then introduced into G27 and K74 *H. pylori*, respectively, by natural transformation. Transformants were selected for on the Columbia blood agar plates supplemented with 25 μg/ml kanamycin, and were further screened on the Columbia blood agar plates supplemented with 5% sucrose to select for a double crossover even that resulted in the deletion of *cagL*. Single colonies of kanamycin-resistant, sucrose-sensitive *H. pylori* were verified by PCR amplification and DNA sequencing of the genomic locus.

### Construction of the human gastrin promoter-luciferase reporter plasmid

Human genomic DNA purified from AGS cells (human gastric adenocarcinoma, ATCC CRL 1739) was used as a template to PCR amplify a segment spanning from −240 to +60 bp of the human gastrin promoter (Shiotani and Merchant, 1995) using *Pfu* polymerase. The sequence of the forward primer was 5′-AAA GAG CTC AGC TGG AGA GCT GCC GCC-3′ and the reverse primer was 5′-CCC CTC GAG CTG CAG AGC TGG GAG GTG TG-3′. The PCR product was then cloned into the pGL3 basic vector (Promega) using the XhoI and SacI restriction sites and the resulting gastrin promoter-luciferase construct, pG240-Luc, was confirmed by DNA sequencing.

### Stable transfection of AGS cells

AGS cells were cultured in DMEM medium (Gibco) supplemented with 10% FBS and 1% penicillin/streptomycin (Gibco) at 37°C in a humidified incubator containing 5% CO_2_. AGS cells were cotransfected with pG240-Luc and pcDNA3 mammalian expression vector (Invitrogen), and transfectants were selected by 400 μg/ml of G418 (Sigma-Aldrich). Cells showing high levels of luciferase activity were selected, pooled, and stored as G240-Luc cells until use.

### *H. pylori* infection and treatment of recombinant proteins

G240-Luc or AGS cells were seeded onto a cell culture plate. After reaching 80% confluence, cells were serum-starved for 24 h, and then either infected with *H. pylori* at a multiplicity of infection (MOI) of 100 or treated with 10 nM of recombinant EGF family proteins (HB-EGF, EGF, and AR). Treatment of 1 μM AG1478 (Calbiochem) was done 30 min prior to *H. pylori* infection or recombinant protein treatment.

### Transient transfection using small interfering RNA (siRNA)

The sequences of siRNA used in this study are listed in Supplementary Table 1. A-Raf and HB-EGF siRNA consisted of pools of 3 and 2 target-specific siRNAs, respectively. TGF-α, AR, and EGF siRNA consisted of 2 target-specific siRNAs each. All other knockdowns were achieved with a single siRNA molecule. G240-Luc or AGS cells were plated in cell culture plates in DMEM supplemented with 10% FBS. After the cells reached 80% confluence, cells were serum-starved for 2 h, and then transfection was carried out to reach a final siRNA concentration of 50 or 100 pM using Lipofectamine 2000 (Invitrogen) according to the manufacturer's instructions. Six hours post transfection, cell culture media was changed to DMEM with 10% FBS. After 48 h of transfection, cells were infected with *H. pylori* at a MOI of 100, for the indicated durations. Specific knockdown of targeted genes was confirmed by Western blot.

### Western blot analysis

Cells were washed with phosphate-buffered saline (PBS) and then lysed with cell lysis buffer (Cell Signaling Technology), as described by the manufacturer. Proteins were separated by sodium dodecyl sulfate polyacrylamide gel electrophoresis (SDS-PAGE) and then transferred to polyvinylidene difluoride (PVDF) membranes (Millipore). After blocking with blocking buffer composed of Tris-buffered saline (TBS) containing 0.1% Tween 20 and 5% skim milk (BD), for 1 h at room temperature, the membranes were incubated with the following antibodies: A-Raf, B-Raf, C-Raf, CagA, and Urease A (1:1,000, Santa Cruz Biotechnology), EGFR, phospho EGFR, Mek1/2, Erk1/2, and phospho Erk1/2 (1:1,000, Cell Signaling Technology), or glyceraldehyde-3-phosphate dehydrogenase (GAPDH) (1:1,000, AbFrontier) overnight at 4°C. Subsequently, membranes were washed three times with TBS containing 0.1% Tween 20 and then incubated with horseradish peroxidase-conjugated Goat anti-rabbit IgG (Santa Cruz Biotechnology) or Goat anti-mouse IgG (Santa Cruz Biotechnology) for 1 h at room temperature. The protein bands were visualized using the enhanced chemiluminescence solution (Advansta) as described by the manufacturer.

### *In vitro* assay of gastrin promoter activity

G240-Luc cells were plated in 12-well plates at a density of 1 × 10^5^ cells per well in 1 ml of DMEM supplemented with 10% FBS. When the cells reached 80% confluence, cells were treated with recombinant proteins, infected with *H. pylori*, or transfected with siRNA as described earlier. Five hours after stimulation, gastrin promoter activity was measured using the luciferase assay.

### Luciferase assay

Cells were washed with PBS and lysed with passive lysis buffer (Promega). Cells were scraped, and centrifuged at 12,000 × *g* for 2 min at 4°C. Luciferase activities were measured with a luciferase assay system (Promega) and a Centro XS^3^ microplate luminometer LB 960 (Berthold Technologies). Total protein in cell lysates was determined by the bicinchoninic acid assay (Pierce Biotechnology) and was used for normalization.

### Quantitative RT-PCR (qRT-PCR)

AGS and GES-1 (Ke et al., 1994) cells were plated in 6-well cell culture plates at a density of 4 × 10^5^ cells per well in 2 ml of DMEM supplemented with 10% FBS. After achieving 80% confluence, cells were treated with recombinant proteins, infected with *H. pylori*, or transfected with siRNA as described earlier. The immortalized human gastric mucosal cell line GES-1 was provided by the Yonsei University College of Medicine, Seoul, Korea. After incubating the cells for the indicated times, total RNA was extracted and converted to cDNA. Expression level of gastrin, HB-EGF, AR, EGF, TGF-α, and 18S rRNA was measured by qRT-PCR. Total RNA was extracted from cultured cells using Trizol reagent (Invitrogen) in accordance with the manufacturer's instructions. One microgram of total RNA was converted to cDNA using the RT premix kit (Bioneer) according to the manufacturer's instructions. qRT-PCR was conducted using a SYBR® Premix Ex *Taq*™ (Takara Bio) and Applied Biosystems 7300 Real-Time PCR System (Applied Biosystems). The specific primers and annealing temperature utilized for qRT-PCR of gastrin, HB-EGF, AR, EGF, TGF-α, and 18S rRNA are listed in Supplementary Table 2.

An absolute quantification method using a standard curve was performed to measure mRNA expression levels of the EGF family members. Each gene of the human EGF family (HB-EGF, EGF, AR, TGF-α, BTC, epigen, and epiregulin) was cloned in a pGEM-T Easy vector to establish a standard curve for each gene. The standard curve equation was used to calculate the absolute mRNA copy number of each target (Chini et al., 2007). Relative quantification method was also performed for gastrin and HB-EGF mRNA expression, in relation to 18S rRNA mRNA expression by the application of the 2^−ΔΔ*Ct*^ analysis method (Livak and Schmittgen, 2001). All experiments were conducted in triplicate.

### Field emission scanning electron microscopy analysis

*H. pylori* and AGS human gastric cells were co-cultured at a MOI of 100 on tissue culture-treated coverslips (BD) for 4 h at 37°C in the presence of 5% CO_2_. Cells were fixed with 2.0% paraformaldehyde, 2.5% glutaraldehyde in phosphate buffered saline (PBS). Coverslips were washed with sodium cacodylate buffer and secondary fixation was performed with 1% osmium tetroxide at room temperature for 90 min. Coverslips were washed with sodium cacodylate buffer and dehydrated with sequential washes of increasing concentrations of ethanol. Samples were then dried at the critical point, mounted onto sample stubs with a carbon tape, sputter-coated with platinum, and viewed with a HITACHI S-4700 FE scanning electron microscope (Hitachi).

### IL-8 Secretion assay

AGS cells were plated in 6-well cell culture plates at a density of 4 × 10^5^ cells per well in 2 ml of DMEM supplemented with 10% FBS. After achieving 80% confluence, cells were serum-starved for 2 h, and then cells were transfected with non-targeting (NT) siRNA (100 pM), or HB-EGF siRNA (50 and 100 pM). Six hours post transfection, cell culture media was changed to DMEM with 10% FBS. After 48 h of transfection, cells were infected with *H. pylori* at a MOI of 100, for 5 h. Five hours post infection, the concentration of IL-8 in cell culture supernatant was measured by enzyme linked immunosorbent assay using Human IL-8 ELISA MAX™(Biolegend), following the manufacturer's instructions.

### Cell elongation assay

AGS cells were plated in 6-well cell culture plates at a density of 4 × 10^5^ cells per well in 2 ml of DMEM supplemented with 10% FBS. After achieving 80% confluence, cells were serum-starved for 2 h, and then cells were transfected with non-targeting (NT) siRNA (100 pM), or HB-EGF siRNA (50 and 100 pM). Six hours post transfection, cell culture media was changed to DMEM with 10% FBS. After 48 h of transfection, cells were infected with *H. pylori* at a MOI of 100, for 5 h. Five hours post infection, cells were fixed with 4% paraformaldehyde. Images of the cells were taken using a CKX41 inverted microscope and DP20 microscope camera (Olympus) under × 200 magnification.

### HA and myc-double tagging of HB-EGF

The double epitopes-tagged proHB-EGF (proHBEGF-HA/Myc) was constructed as described previously (Gechtman et al., 1999). The amino-terminal region of the protein was fused with an HA epitope and the carboxy-terminal region with a Myc epitope using SOE PCR using *Pfu* DNA polymerase and HB-EGF cDNA as a template. Briefly, two DNA oligonucleotide primers sets were used as follows: the forward primer set, HB-EGF(F)-HindIII (5′-CCC AAG CTT GCA TGA AGC TGC TGC CGT CG-3′) and HA(F), which was designed to contain HA overlapping sequence (5′-CTA CCC ATA CGA CGT CCC AGA CTA CGC TAC ACC AAG CAA GGA GGA G-3′), and the reverse primer set of HA(R), which was designed to contain HA overlapping sequence (5′-GCG TAG TCT GGG ACG TCG TAT GGG TAA GTG ACT CTC AAA AGG TCC AG-3′) and HB-EGF(R)-XbaI, which contained the Myc epitope sequence: 5′-GCT CTA GAT CAC AGA TCC TCT TCT GAG ATG AGT TTT TGT TCG TGG GAA TTA GTC ATG CC-3′). The HB-EGF(F)-HindIII and (R)-XbaI primers were designed to include HindIII and XbaI restriction enzyme sites, respectively. The amino and carboxy terminal regions were amplified individually using HB-EGF(F)-*Hin*d III and HA(R), and HA(F) and HB-EGF(R)-XbaI, respectively. These regions were then fused together by SOE PCR. The resulting fused PCR product was cloned into the pGEM-T Easy vector and sequenced for confirmation. For expression studies, the proHBEGF-HA/Myc gene was excised from the pGEM-T Easy vector by HindIII and XbaI double digestion and then subcloned into the HindIII and XbaI sites of the pcDNA3, resulting in the plasmid pHB-EGF.

### Preparation of uncleavable proHBEGF-HA/Myc

A non-cleavable form of proHBEGF-HA/Myc (Uc-proHBEGF-HA/Myc) was created by mutation of the cleavage site in the epitope-tagged proHBEGF-HA/Myc; “L^148^PVENR^153^” was replaced with “STLLPT” (Hirata et al., 2001). Briefly, this was accomplished by *Pfu* polymerase amplification of the amino and carboxy termini of HB-EGF using primers that incorporated the desired change. The primer set for the amino terminus were as follows: a forward primer (HB-EGF-F); 5′-CCC AAG CTT GCA TGA AGC TGC TGC CGT CG-3′, corresponding to the 5′ end of the full-length HB-EGF open reading frame, and a reverse primer (P1-R), 5′-AGT TGG GAG AAG TGT AGA GCT CAG CCC ATG ACA CCT CTC TC-3′, containing the desired mutation in the cleavage site. The carboxy primer pair were as follows: a forward primer (P2-F), 5′-TCT ACA CTT CTC CCA ACT TTA TAT ACC TAT GAC CAT ACA ACT ATC C-3′, including sequence to modify the cleavage site, and a reverse primer (HB-EGF-R), 5′-GCT CTA GAT CAC AGA TCC TCT TCT GAG ATG AGT TTT TGT TCG TGG GAA TTA GTC ATG CC-3′, which was designed to be complementary to the nucleotide sequence of a Myc tag followed by the 3′ end of the full-length HB-EGF. While the P1-R and P2-F primers contained overlapping sequences to modify the cleavage site, the HB-EGF-F and R primers included HindIII and XbaI restriction enzyme sites, respectively. Again, the amino and carboxy terminus were fused by SOE PCR and the fused PCR product was cloned into the pGEM-T Easy vector. The correct sequence of Uc-proHBEGF-HA/Myc was confirmed by DNA sequence analysis and was then subcloned into the HindIII and XbaI site of the pcDNA3, resulting in the plasmid pUcHB-EGF.

### Ectodomain shedding of HB-EGF

Either pHB-EGF or pUcHB-EGF was transiently transfected into AGS cells using Lipofectamine 2000. Six hours post-transfection, cell culture media was changed to DMEM with 10% FBS. After 48 h of transfection, cells were infected with *H. pylori* for 5 h at a MOI of 100 or treated with 1 μM 12-*O*-Tetradecanoylphorbol-13-acetate (TPA) for 30 min. Shedding of HB-EGF was examined using Western blot and immunofluorescence analysis.

### Western blot analysis of HB-EGF shedding

After the pHB-EGF or pUcHB-EG-transfected cells were infected with *H. pylori* or treated with TPA, conditioned media (CM) were collected from the cells. CM were concentrated using Amicon® Ultra-0.5 centrifugal filter (Millipore) according to the manufacturer's instructions. Concentrated CM was tested for the presence of the HA-tagged soluble HB-EGF ectodomain by western blotting with anti-HA antibody (1:2,500, Santa Cruz Biotechnology). To detect the remaining transmembrane forms and tail fragments of HB-EGF, cells were washed with PBS, and then lysed with cell lysis buffer, as described by the manufacturer. Fifty micrograms of total cell lysates were subjected to SDS-PAGE on 10–20% acrylamide gels and transferred onto a PVDF membrane in 40 mM CAPS buffer, pH 10.5, in 20% methanol. Western blot analysis was performed with anti-Myc antibody (1:2,500, Santa Cruz Biotechnology). The antibodies were visualized with horseradish peroxidase conjugated goat anti-rabbit IgG using ECL plus western blotting detection reagents (Amersham). Band intensities were measured using ImageJ software version 1.47 (National Institutes of Health).

### Immunofluorescence analysis for proHB-EGF shedding

AGS cells grown on 12-mm-diameter glass coverslips were washed with ice-cold PBS, and fixed for 15 min at room temperature using 4% paraformaldehyde diluted in PBS. Slides were washed twice with PBS for 5 min and incubated in blocking solution (1% bovine serum albumin and 0.02% sodium azide in PBS) for 30 min at room temperature. The blocking mixture was aspirated and cells were incubated overnight at 4°C with rabbit anti-HA (1:100) primary antibody. The slides were then washed three times with PBS for 5 min and incubated for 1 h at room temperature with goat anti-rabbit Alexa488 (1:100, Molecular Probes) secondary antibodies diluted in the blocking solution. The slides were washed again before counterstaining with Alexa Fluor 594-conjugated actin phalloidin. Following three washes with PBS, the samples were mounted with Vectashield (Vector Laboratories) and visualized with a Zeiss LSM700 confocal microscope (Carl Zeiss). Green and red fluorescence were measured using ImageJ software version 1.47.

### Statistical analysis

Data are presented as the means ± standard deviation. Results were analyzed using one-way analysis of variance with SPSS software version 23 (IBM). Multiple comparisons were performed using Tukey's method. A *P*-value less than 0.05 was considered to be statistically significant.

## Results

### *H. pylori* infection induces gastrin mRNA expression

**Figure 1:**
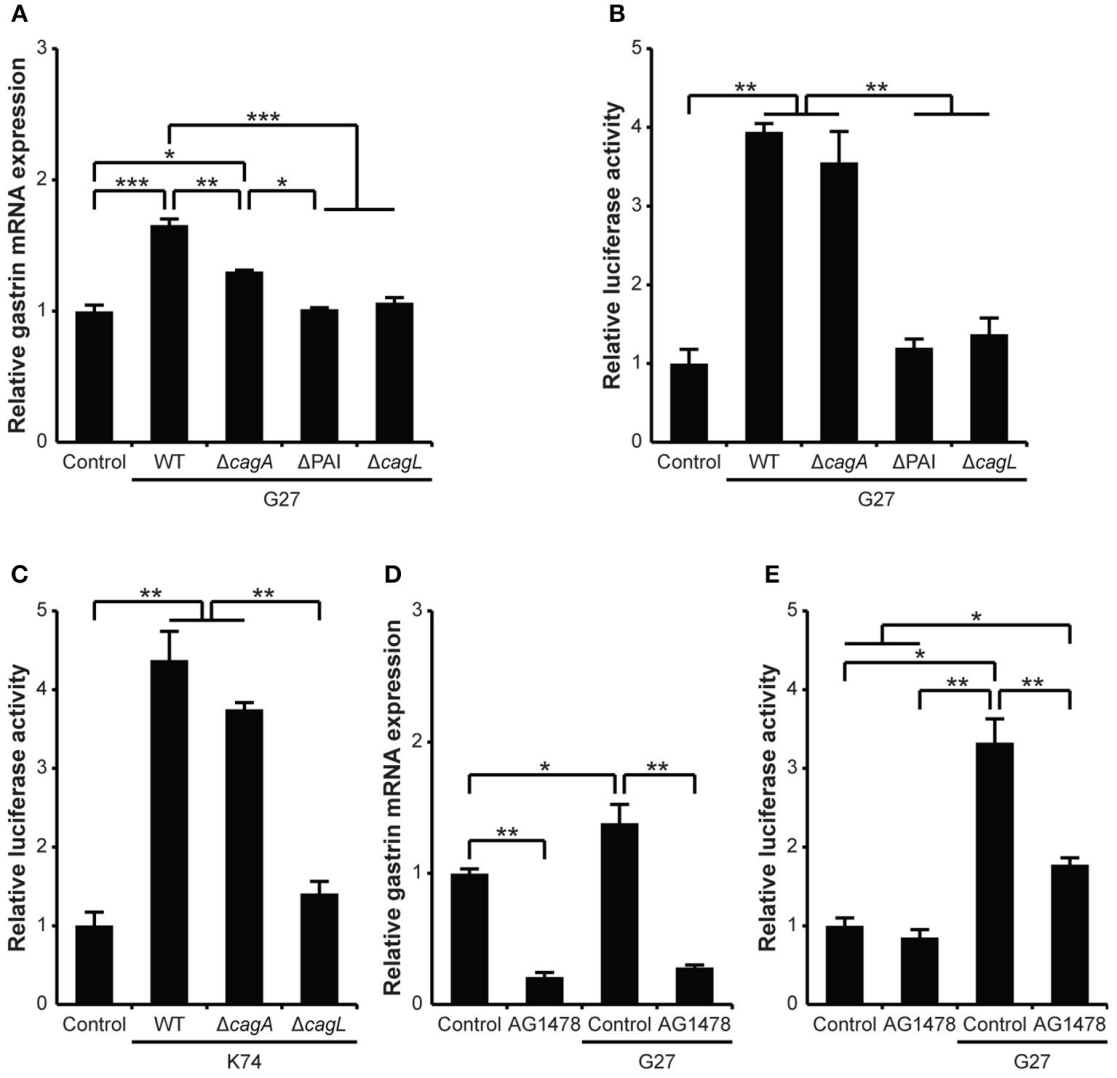
*H. pylori* infection increases gastrin expression in gastric epithelial cells through the EGF receptor. **(A)** Gastrin mRNA expression was measured by qRT-PCR using RNA isolated at 5 h after AGS cells were infected with *H. pylori* G27 WT or its isogenic mutant strains: G27Δ*cagA*, G27ΔPAI, and G27Δ*cagL*. The expression of gastrin mRNA was relatively quantified using the 2^−ΔΔCt^ method. The expression of 18S rRNA was used as a reference. **(B)** G240-Luc cells were infected with G27 WT or its isogenic mutant strains: G27Δ*cagA*, G27ΔPAI, and G27Δ*cagL*. **(C)** G240-Luc cells were infected with Korean clinical isolate K74 or its isogenic mutant strains: K74Δ*cagA* and K74Δ*cagL*. **(D)** G240-Luc cells were pretreated with AG1478 (1 μM) for 30 min and infected with G27 WT. Five hours post infection, luciferase activity was measured and presented as a relative ratio to that of uninfected control cells. **(E)** Gastrin mRNA expression was measured by qRT-PCR using RNA isolated at 5 h after AGS cells were infected with *H. pylori* G27 WT, with or without pretreatment of AG1478 (1 μM) for 30 min. Each result is presented as mean values ± standard deviation (*SD*) for three separate experiments, each of which was performed in triplicate. ^*^P < 0.05, ^**^P < 0.01, ^***^P < 0.001.

To examine the ability of *H. pylori* to regulate gastrin expression, we infected AGS epithelial cells with wild-type (WT) G27 *H. pylori* and measured gastrin mRNA levels by quantitative reverse transcription PCR (qRT-PCR) at 2.5 and 5 h post-infection. Uninfected cells were maintained as a control for the basal level of gastrin mRNA expression. As shown in Supplementary Figure 1, G27 significantly induced gastrin mRNA expression at 5 h as compared to control cells. To further monitor *H. pylori*-induced gastrin expression, AGS cells and G240-Luc cells containing a gastrin promoter-luciferase fusion were infected with G27 WT or various isogenic mutant strains. As shown in Figures 1A,B, G27 significantly induced gastrin mRNA expression by as much as 1.7-fold as compared to control cells at 4 h post-infection and furthermore induced gastrin promoter activity by as much as 4-fold. Of note, gastrin promoter activity was not induced significantly at 3 h post-infection (data not shown). Gastrin mRNA expression induced by G27Δ*cagA* was also significantly higher than the control, but lower than that induced by G27 WT. However, the increase in gastrin promoter activity induced by G27Δ*cagA* was similar to the promoter activation induced by G27 WT. Conversely, strains G27Δ*cagL* or G27ΔPAI failed to induce gastrin mRNA expression or promoter activity.

To determine whether the induction of gastrin mRNA was a specific phenomenon of the Western G27 *H. pylori* strain, we also used the East Asian K74 *H. pylori* strain, which is an *H. pylori* clinical isolate originally obtained from Korea (Jones et al., 2009). Similar to the G27 strain results, K74 and the K74Δ*cagA* mutant significantly induced gastrin promoter activity, while the strain lacking *cagL* (K74Δ*cagL*) failed to induce gastrin promoter activity (Figure 1C). Thus, induction of gastrin mRNA appears to be a general phenomenon of Western and East Asian *H. pylori* strains.

Because the T4SS apparatus is known to be critical for *H. pylori*-induced gastrin expression (Wiedemann et al., 2012), we wished to analyze the presence of the T4SS apparatus upon interaction of *H. pylori* with the surface of the AGS cells. To accomplish this, we used high resolution field emission scanning electron microscopy. We identified the T4SS apparatus of G27, G27Δ*cagA*, K74, and K74Δ*cagA*. However, as expected the T4SS apparatus of G27ΔPAI, G27Δ*cagL and* K74Δ*cagL* were no longer present (Supplementary Figure 2 and data not shown). *En masse*, these data indicate that *H. pylori*-mediated gastrin promoter activation requires the T4SS apparatus of *H. pylori*, but does not require the CagA effector protein.

### Activation of the EGF receptor is required for full *H. pylori*-induced gastrin promoter activation

The binding of ligands to the EGF receptor is the initial event required for growth factor(s)-mediated Erk pathway activation. Therefore, to determine whether activation of the EGF receptor mediates *H. pylori*-induced gastrin promoter activation, we used an EGF receptor kinase inhibitor, AG1478, to block this activation step. Treatment of uninfected cells with AG1478 resulted in a decrease in gastrin mRNA expression but no significant change in basal level activity of the gastrin promoter (Figures 1D,E). For G27 WT infection, pretreatment of cells with AG1478 significantly reduced both *H. pylori*-induced gastrin mRNA expression and gastrin promoter activation. It is worth noting that AG1478 did not completely block *H. pylori*-induced gastrin promoter activation. Since the EGF receptor is upstream of the MAPK pathway, we also examined *H. pylori*-induced Erk activation. AGS cells were infected with G27 in the absence or presence of AG1478 pretreatment and Erk activation was measured by Western blot analysis. *H. pylori* CagA expression was detected regardless of AG1478 pretreatment after G27 WT infection. Furthermore, G27 infection induced Erk activation; Erk activation was prominent 30 and 60 min post infection and persisted up to 480 min (Supplementary Figure 3A, left panel). Erk activation was remarkably reduced by AG1478 especially at later time points (Supplementary Figure 3A, right panel). This suggests that AG1478 inhibits *H. pylori*-induced Erk activation. *En masse*, these results suggest that activation of the EGF receptor is required for full *H. pylori*-induced gastrin expression.

### *H. pylori* infection induces mRNA expression of EGF family members

**Figure 2:**
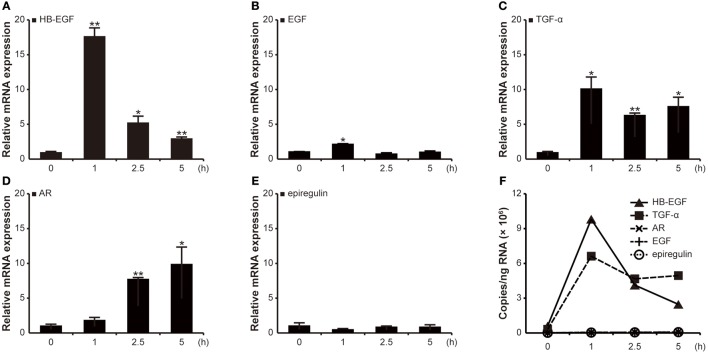
Relative and absolute quantification of transcription levels of EGF family members in AGS cells after *H. pylori* infection. AGS cells were infected with G27 WT for the indicated durations. Transcriptions of HB-EGF, TGF-α AR, EGF, and epiregulin were relatively quantified by qRT-PCR. **(A–E)** The results were expressed as relative transcription levels of each EGF family gene to those of the uninfected cells. Each result is presented as mean values ± *SD* of three independent experiments performed in triplicate. ^*^P < 0.05, ^**^P < 0.01. **(F)** The absolute levels of transcription of all EGF family genes are shown together.

Since activation of the EGF receptor is important for *H. pylori*-induced gastrin promoter activation, the absolute levels of mRNA expression were next determined for the EGF family members (HB-EGF, AR, EGF, TGF-α, BTC, epigen, and epiregulin) using qRT-PCR at 1, 2.5, and 5 h post-infection. As shown in Figure 2A, the G27 strain induced an 18-fold increase in HB-EGF mRNA copies at 1 h as compared to a basal expression level of HB-EGF mRNA in control cells. Similarly, EGF and TGF-α mRNAs were maximally increased at 1 h: 2- and 10-fold, respectively (Figures 2B,C). Conversely, the AR mRNA levels showed the highest levels at 5 h (10-fold increase, Figure 2D) and mRNA expressions of epiregulin was not significantly changed (Figure 2E). The results of BTC and epigen were similar to that of epiregulin (data not shown). The absolute quantifications of mRNA copies for HB-EGF and TGF-α were extremely high after *H. pylori* infection (Figure 2F).

### Role of EGF family members in *H. pylori*-induced gastrin expression

**Figure 3:**
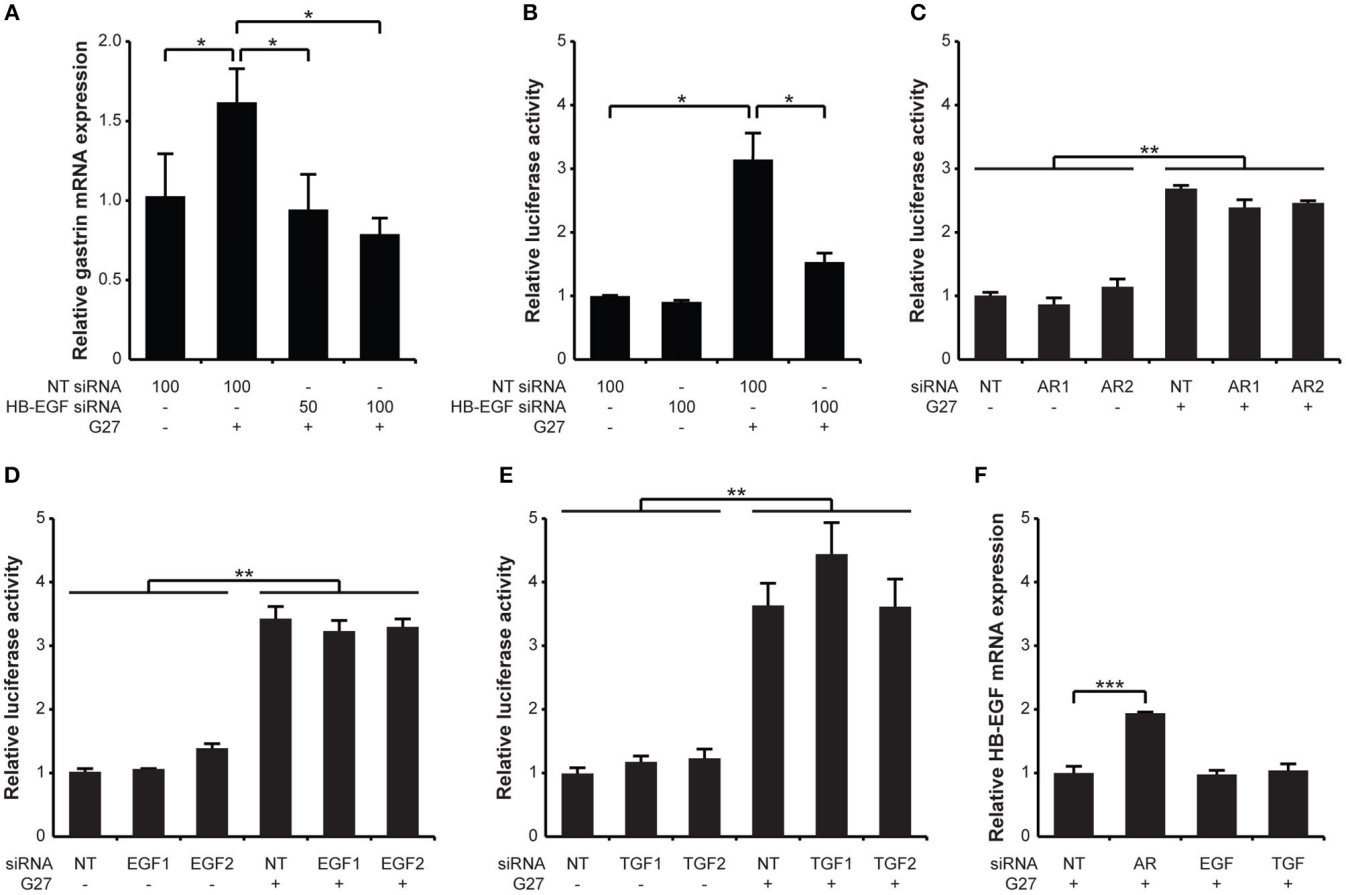
Specific siRNA knockdown of HB-EGF inhibits *H. pylori*-induced transcription and promoter activity of gastrin. **(A)** AGS and **(B)** G240-Luc cells were transfected with non-targeting (NT) or HB-EGF siRNA at the indicated concentrations (50 or 100 pM). **(C–E)** AGS cells were transfected with 100 pM of siRNAs targeting each AR, EGF, or TGF-α. **(F)** AGS cells were transfected with 100 pM of siRNA (NT or EGF), or with a 1:1 mixture of 2 siRNAs (50 pM each, TGF-α or AR). Forty-eight hours post transfection, the cells were infected with *H. pylori* G27 for 5 h. Transcription of gastrin or HB-EGF was relatively quantified to that of 18S rRNA by qRT-PCR. Promoter activity was presented as a relative ratio of the luciferase activity level to that of the NT siRNA-treated cells. Each result is presented as mean values ±*SD* of three separate experiments performed in triplicate. ^*^P < 0.05, ^**^P < 0.01, ^***^P < 0.001.

The involvement of HB-EGF, TGF-α, AR, and EGF in gastrin expression was investigated using specific siRNA knockdown of each in either AGS or G240-Luc cells followed by *H. pylori* infection. Knockdown of HB-EGF in AGS cells significantly reduced gastrin mRNA expression induced by *H. pylori* infection (Figure 3A). Similarly, significant reductions in *H. pylori*-induced gastrin mRNA promoter activation were observed when HB-EGF was knocked down in the G240-Luc cells (Figure 3B). In contrast, knockdown of AR, EGF, or TGF-α did not result in any significant reduction in *H. pylori*-induced gastrin promoter activity (Figures 3C–E). Of note, knockdown of HB-EGF in AGS cells did not result in any significant reduction in *H. pylori*-induced interleukin-8 (IL-8) expression or cell elongation (Supplementary Figures 4, 5), suggesting a specific role for HB-EGF in gastrin expression. We also examined the effect of siRNA knockdown of EGF, TGF-α, or AR on HB-EGF mRNA expression (Figure 3F). Knockdown of AR resulted in a 2-fold increase in *H. pylori*-induced HB-EGF mRNA expression, while knockdown of EGF and TGF-α showed no effect on HB-EGF mRNA expression. *En masse*, among the EGF family members, only knockdown of HB-EGF reduced *H. pylori*-induced gastrin expression, suggesting that HB-EGF may play a critical role in gastrin expression.

### HB-EGF can stimulate gastrin mRNA expression

**Figure 4:**
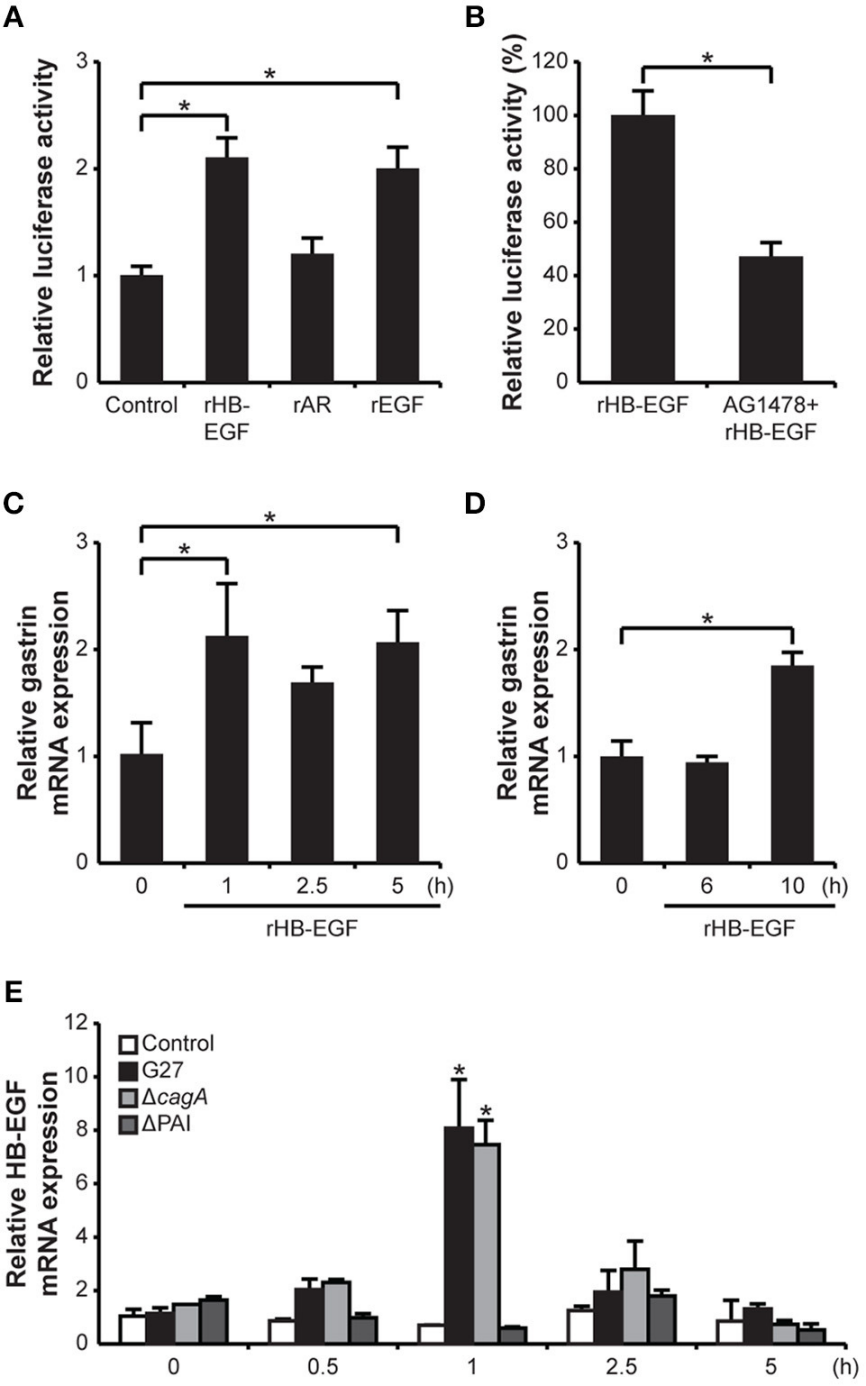
Effect of HB-EGF on transcription and promoter activity of gastrin, and effect of *H. pylori* on transcription of HB-EGF. **(A)** G240-Luc cells were stimulated with 10 nM of each recombinant HB-EGF (rHB-EGF), AR (rAR), or EGF (rEGF) for 5 h and gastrin promoter activity was measured by luciferase assay. **(B)** G240-Luc cells were pretreated with EGF receptor kinase inhibitor AG1478 (1 μM) for 30 min and subsequently stimulated with rHB-EGF. Gastrin promoter activity was measured by luciferase assay. **(C)** AGS cells or **(D)** GES-1 cells were stimulated with rHB-EGF for the indicated durations. Transcription levels of gastrin were relatively quantified by qRT-PCR. **(E)** After *H. pylori* infection for the indicated durations, HB-EGF mRNA expression was measured by qRT-PCR. Each result is presented as a fold change relative to the control ±*SD*. ^*^P < 0.05.

Since the results obtained from the siRNA knockdown of HB-EGF suggested that HB-EGF is directly involved in *H. pylori*-induced gastrin expression, we next investigated whether treatment with recombinant protein could recapitulate changes in gastrin mRNA expression. To accomplish this, G240-Luc cells were treated with recombinant HB-EGF (rHB-EGF), AR (rAR), or EGF (rEGF). As shown in Figure 4A, rHB-EGF induced significant gastrin promoter activation; levels were similar to that seen with the rEGF, which is known to stimulate gastrin expression (Godley and Brand, 1989). However, rAR did not induce gastrin promoter activation. To confirm that the increases in gastrin promoter activity induced by rHB-EGF were due to activation of the EGF receptor, G240-Luc cells were pretreated with the EGF receptor inhibitor AG1478 prior to treatment with rHB-EGF. As shown in Figure 4B, blockage of the EGF receptor completely abrogated induction of gastrin expression. To further confirm the role of rHB-EGF in gastrin mRNA expression, we also assessed induction in AGS and GES-1 cells. Treatment with rHB-EGF induced significant gastrin mRNA expression as early as 1 h-post treatment in AGS cells, but required 10 h in GES-1 cells (Figures 4C,D). These data strongly suggest that mature HB-EGF may play a role in *H. pylori*-induced gastrin expression through transactivation of the EGF receptor.

### *H. pylori*-mediated HB-EGF induction is T4SS-dependent

To determine which virulence factors of *H. pylori* were important for induction of HB-EGF expression, mRNA levels were temporally (0.5, 1, 2.5, and 5 h post-infection) examined after *H. pylori* infection with G27 or the G27Δ*cagA* and G27ΔPAI mutant strains. Both G27 and G27Δ*cagA* significantly induced HB-EGF mRNA expression at 1 h, whereas G27ΔPAI didn't induce HB-EGF mRNA (Figure 4E). Next, to assess the activation of EGFR by *H. pylori* infection, phosphorylation of EGFR was measured by Western blot analysis. Expression and Phosphorylation of EGFR was slightly increased by G27 WT (Supplementary Figure 3B). *En masse*, these data indicate that *H. pylori*-mediated HB-EGF induction requires the T4SS apparatus of *H. pylori*.

### *H. pylori*-induced HB-EGF ectodomain shedding is an important process for gastrin promoter activation

**Figure 5:**
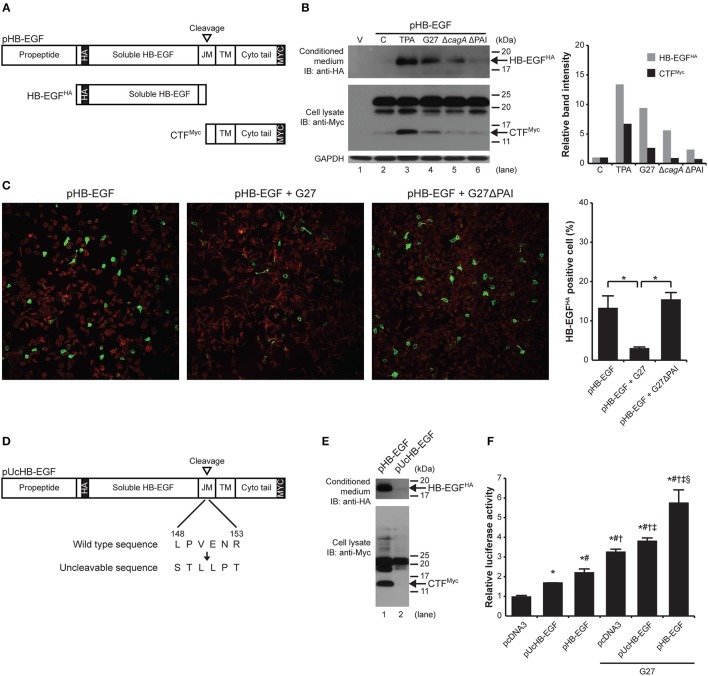
*H. pylori*-induced HB-EGF shedding is important for gastrin expression. **(A,D)** Schematic diagrams of double epitope-tagged membrane-anchored proHBEGF-HA/Myc and Uncleavable proHBEGF-HA/Myc constructs, pHB-EGF, and pUcHB-EGF, respectively. **(B)** AGS cells were transfected with pcDNA3 (lane 1) or with pHB-EGF (lanes 2–6). Cells expressing proHBEGF-HA/Myc were treated with 1 μM TPA for 30 min, or infected with G27 WT, G27Δ*cagA*, or G27ΔPAI for 4 h. Existence of shed HB-EGF^HA^ in concentrated conditioned medium (upper panel) and CTF^Myc^ in cell lysate (lower panel) were examined by Western blot with anti-HA and anti-Myc antibody, respectively. Intensities of each HB-EGF^HA^ or CTF^Myc^ band was measured and normalized to intensity of GAPDH. Intensities of each band were then compared to that of the control, and presented as relative band intensity. **(C)** AGS cells transfected with pHB-EGF were infected with G27 WT or G27ΔPAI for 4 h and proHBEGF-HA/Myc was observed using confocal microscopy. proHBEGF-HA/Myc and actin were stained with green and red fluorescence, respectively. Each image is representative of three independent experiments. The proportion of HB-EGF^HA^ positive cells was obtained by calculating ratio between the green-fluorescent area and the total fluorescent area. The average proportion of HB-EGF^HA^ positive cells was derived from two independent experiments. **(E)** AGS cells were transfected with pHB-EGF (lane 1) or with pUcHB-EGF (lane 2), and treated with 1 μM TPA for 30 min. Concentrated conditioned medium (upper panel) and cell lysate (lower panel) were analyzed by Western blot to detect HB-EGF^HA^ and CTF^Myc^, respectively. **(F)** G240-Luc cells were transfected with pcDNA3, pUcHB-EGF, or pHB-EGF, and subsequently infected with G27 for 4 h. Gastrin promoter activity was measured by luciferase assay. ^*^, #, †, ‡, and § indicates statistically significant difference (*P* < 0.05) compared to pcDNA3, pUcHB-EGF, pHB-EGF, pcDNA3 + G27, and pUcHB-EGF + G27, respectively. Luciferase activity was presented as a relative ratio to that of pcDNA3-transfected, uninfected control cells. The mean values ±*SD* of three separate experiments performed in triplicate are shown.

Given that *H. pylori* induced HB-EGF mRNA expression, which would lead to increased production of proHB-EGF, and given that mature HB-EGF induced gastrin mRNA expression, we next investigated the mechanism leading to ectodomain shedding of proHB-EGF to become mature HB-EGF. In previous studies, endogenous HB-EGF protein was shown to be present as different HB-EGF forms due to differential N-terminal processing or due to other modifications of mature HB-EGF (Higashiyama et al., 1992; Iwamoto et al., 1994; Goishi et al., 1995). Thus to overcome issues related to detection of the HB-EGF protein, we labeled HB-EGF with an extracellular HA tag and a cytoplasmic Myc tag. AGS cells were transiently transfected with a pHB-EGF construct engineered to overexpress proHBEGF-HA/Myc. This construct was designed to monitor cleavage at the ectodomain (shedding) via fusion of the hemagglutinin (HA) tag on the extracellular domain (Figure 5A). Similarly, the portion of the protein remaining in the membrane could be monitored via the Myc tag attached to the cytoplasmic domain. In the upper panels of Figures 5B,E, HB-EGF was prepared from the conditioned media of the co-culture system. Thus, the released ~18-kDa mature HB-EGF (HB-EGF^HA^) could only be detected with antibody against the HA tag; the ~22-kDa whole HB-EGF (proHB-EGF) and cleaved ~14-kDa HB-EGF cytoplasmic tail fragment (CTF^Myc^) couldn't be detected. In contrast, in the lower panels of Figures 5B,E, HB-EGF was prepared from cell lysate from the co-culture system. Thus, only proHB-EGF as well as cleaved CTF^Myc^ could be detected; released HB-EGF^HA^ couldn't be detected. As a positive control, treatment of AGS cells with 1 μM TPA for 30 min resulted in the rapid release of HB-EGF^HA^ (13.4-fold) into the conditioned medium, and the appearance of CTF^Myc^ (6.7-fold) compared with the control (Figure 5B, lane 3 vs. 2). Infection with G27 *H. pylori* also resulted in release of HB-EGF^HA^ (9.4-fold) and appearance of CTF^Myc^ (2.6-fold) at 4 h as compared with the control (Figure 5B, lanes 4 vs. 2). Infection with G27Δ*cagA* also resulted in release of HB-EGF^HA^ (5.6-fold) although the band intensity of HB-EGF^HA^ was decreased as compared to G27. Infection with G27ΔPAI resulted in less release of HB-EGF^HA^ (2.4-fold) as compared to G27 and G27Δ*cagA*. The levels of CTF^Myc^ in G27Δ*cagA* and ΔPAI were 0.9- and 0.7-fold, respectively, similar to the control (Figure 5B, lanes 5 and 6 vs. 2). The shedding of cell-associated proHB-EGF following infection with G27 or G27ΔPAI *H. pylori* strains was further monitored by confocal microscopy and staining for proHB-EGF^HA^ (green fluorescence) and actin (red fluorescence). The level of green fluorescence of proHB-EGF^HA^ was dramatically decreased upon G27 *H. pylori* infection (3.1% positive), but not by G27ΔPAI infection (15.5%), compared with a control with pHB-EGF (13.3%; Figure 5C). Thus, consistent with the data in Figure 5B, G27 *H. pylori* induced HB-EGF shedding in a PAI-dependent manner. Western blot analysis (Supplementary Figure 3B) showed that the phosphorylation of the EGF receptor was more prominent with the WT infection than in the control, or G27Δ*cagA* and ΔPAI infections, suggesting that G27 *H. pylori* infection indeed transactivates the EGF receptor. *En masse*, these data indicate that *H. pylori* induces the HB-EGF ectodomain shedding via the T4SS apparatus and probably subsequently transactivates the EGF receptor. Furthermore, though not required for HB-EGF shedding, CagA plays a role in maximal shedding.

In order to determine whether *H. pylori*-induced HB-EGF ectodomain shedding is an important process for induction of gastrin expression, we next directly investigated the requirement of ectodomain shedding for gastrin promoter activation using an uncleavable (Uc) form of HB-EGF (pUcHB-EGF) (Figure 5D). TPA treatment of G240-Luc cells bearing pHB-EGF or pUcHB-EGF verified that the pUcHB-EGF construct results in an uncleavable form of the protein (Figure 5E). Uninfected G240-Luc cells that were transiently transfected with pUcHB-EGF showed significantly higher gastrin promoter activity than G240-Luc cells transfected with pCDNA3. Additionally, the G240-Luc cells with pHB-EGF showed significantly higher gastrin promoter activity than those with pUCHB-EGF. As expected, infection with G27 significantly induced gastrin promoter activity in the G240-Luc cells carrying pcDNA3, and G27 infection of the G240-Luc cells carrying either pUCHB-EGF or pHB-EGF resulted in significantly higher levels of induction than in the G240-Luc cells carrying pcDNA3. More importantly, WT infection of G240-Luc cells carrying cleavable HB-EGF resulted in a significantly higher level of gastrin promoter activity than seen with the G240-Luc cells carrying the UcHB-EGF (Figure 5F). This finding suggests that *H. pylori*-induced HB-EGF ectodomain shedding is an important process for induction of gastrin expression.

### C-Raf is important for *H. pylori*-induced gastrin promoter activity

**Figure 6:**
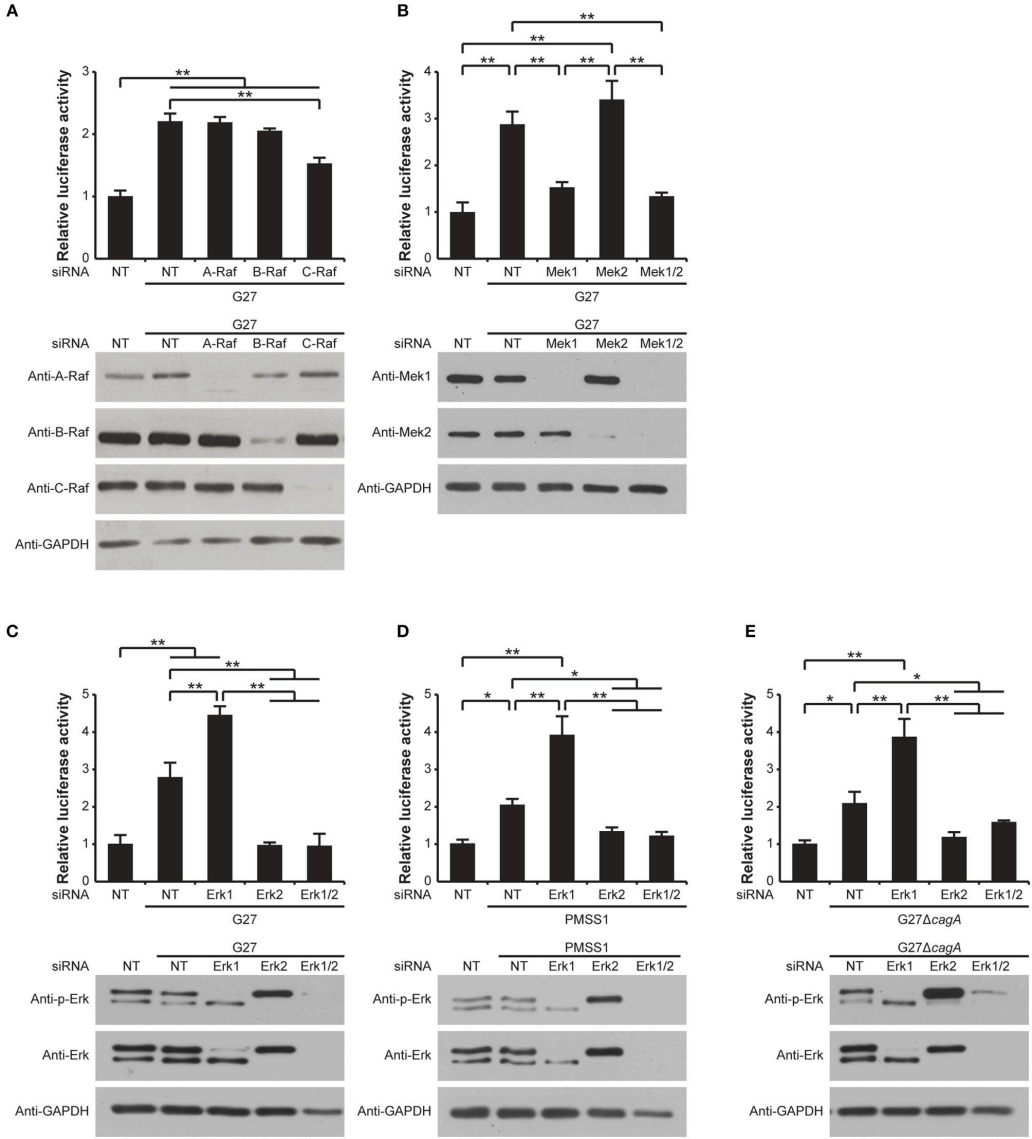
Role of Raf, Mek, and Erk isoforms in the MAPK pathway in *H. pylori*-induced gastrin promoter activation. (**A–E**, upper panels) G240-Luc cells were transfected with 100 pM of NT siRNA, **(A)** A-Raf, B-Raf, or C-Raf siRNA, **(B)** Mek1, Mek2, or both Mek1 and Mek2 (Mek1/2) siRNA, and **(C–E)** Erk1, Erk2, or both Erk1 and Erk2 (Erk1/2) siRNA. Forty-eight hours post transfection, cells were infected with **(A–C)** *H. pylori* strain G27, **(D)** PMSS1, or **(E)** G27Δ*cagA* for 5 h. Luciferase activity is presented as a relative ratio to that of NT siRNA-transfected cells. The mean values ±*SD* of three separate experiments performed in triplicate are shown. ^*^P < 0.05, ^**^P < 0.001. (**A–E**, lower panels) Knockdown of **(A)** A-Raf, B-Raf or C-Raf, **(B)** Mek1 or Mek2, and **(C–E)** Erk1 or Erk2 were confirmed by Western blot. GAPDH was used as a loading control.

In order to further delineate the mechanism leading to induction of gastrin promoter activity after transactivation of the EGF receptor via *H. pylori*-induced HB-EGF expression and shedding, we next investigated the involvement of each Raf isoform in gastrin promoter activity. This was accomplished using specific siRNA knockdown of A, B, or C-Raf in the G240-Luc cells followed by *H. pylori* infection. While knockdown of A-Raf or B-Raf did not result in any reduction in *H. pylori*-induced gastrin promoter activity, a significant reduction in *H. pylori*-mediated gastrin promoter activation was observed when C-Raf was knocked down (Figure 6A, upper panel). The knockdown of each target protein by siRNA treatment was confirmed by Western blot analysis (Figure 6A, lower panel). These data suggest that C-Raf is a mediator of *H. pylori*-induced gastrin promoter activity.

### Mek1 plays a major role in *H. pylori*-induced gastrin promoter activity

Mek1 and Mek2 are known to be downstream substrates of the Raf proteins. We therefore next examined the role of Mek1 and Mek2 on *H. pylori*-induced gastrin promoter activity. To accomplish this, G240-Luc cells were treated with a specific Mek1 or Mek2 siRNA, or a combination of the two siRNAs. While siRNA knockdown of Mek2 did not affect the *H. pylori*-induced gastrin promoter activity compared to the non-targeting siRNA-treated control cells, siRNA knockdown of Mek1 significantly reduced the gastrin promoter activity (Figure 6B, upper panel). Similarly, siRNA knockdown of both Mek1 and Mek2 resulted in a significant reduction in the *H. pylori*-induced gastrin promoter activity to a level comparable to that of the Mek1 siRNA alone. The knockdown of each target protein by siRNA treatment was confirmed by Western blot analysis (Figure 6B, lower panel). These data indicate that Mek1 plays a major role in *H. pylori*-induced gastrin promoter activity, while Mek2 is dispensable.

### Erk1 and Erk2 play different roles in *H. pylori*-induced gastrin promoter activity

Erk is the only known downstream target of Mek. We therefore next investigated the roles of Erk1 and Erk2 on *H. pylori*-mediated gastrin promoter stimulation. G240-Luc cells were treated with siRNAs specific for Erk1 and Erk2, alone or together and then infected with G27. Western blot analysis against phosphorylated or total Erk1 and Erk2 clearly showed the expected specific knockdowns by the respective siRNA treatments (Figure 6C, lower panel). Whereas a significant reduction in *H. pylori*-mediated gastrin promoter activity was observed with siRNA knockdown of Erk2, siRNA knockdown of Erk1 resulted in a significant enhancement in *H. pylori*-mediated gastrin promoter activity (Figure 6C, upper panel). Moreover, siRNA knockdown of both Erk1 and Erk2 significantly reduced the G27-induced gastrin promoter activity to the same level as siRNA knockdown of Erk2 alone (Figure 6C, upper panel).

To determine whether this result was conserved across *H. pylori* strains, these assays were repeated using the *H. pylori* strain PMSS1. PMSS1-induced gastrin promoter activity showed identical patterns to those induced by G27 (Figure 6D). Thus, the differential roles of the Erk isoforms on *H. pylori*-induced gastrin promoter activity appear to be a general phenomenon that is conserved across strains.

Next, since CagA induces sustained Erk activation (Higashi et al., 2004), the G27Δ*cagA* isogenic mutant strain was used to determine whether CagA had any effect on the differential roles of the two Erk isoforms on gastrin promoter activity. G27Δ*cagA* induced gastrin promoter activity identical to the pattern seen with the G27 WT (Figure 6E). These data suggest that the differential roles of the Erk isoforms on *H. pylori*-mediated gastrin promoter activity are CagA-independent.

## Discussion

**Figure 7:**
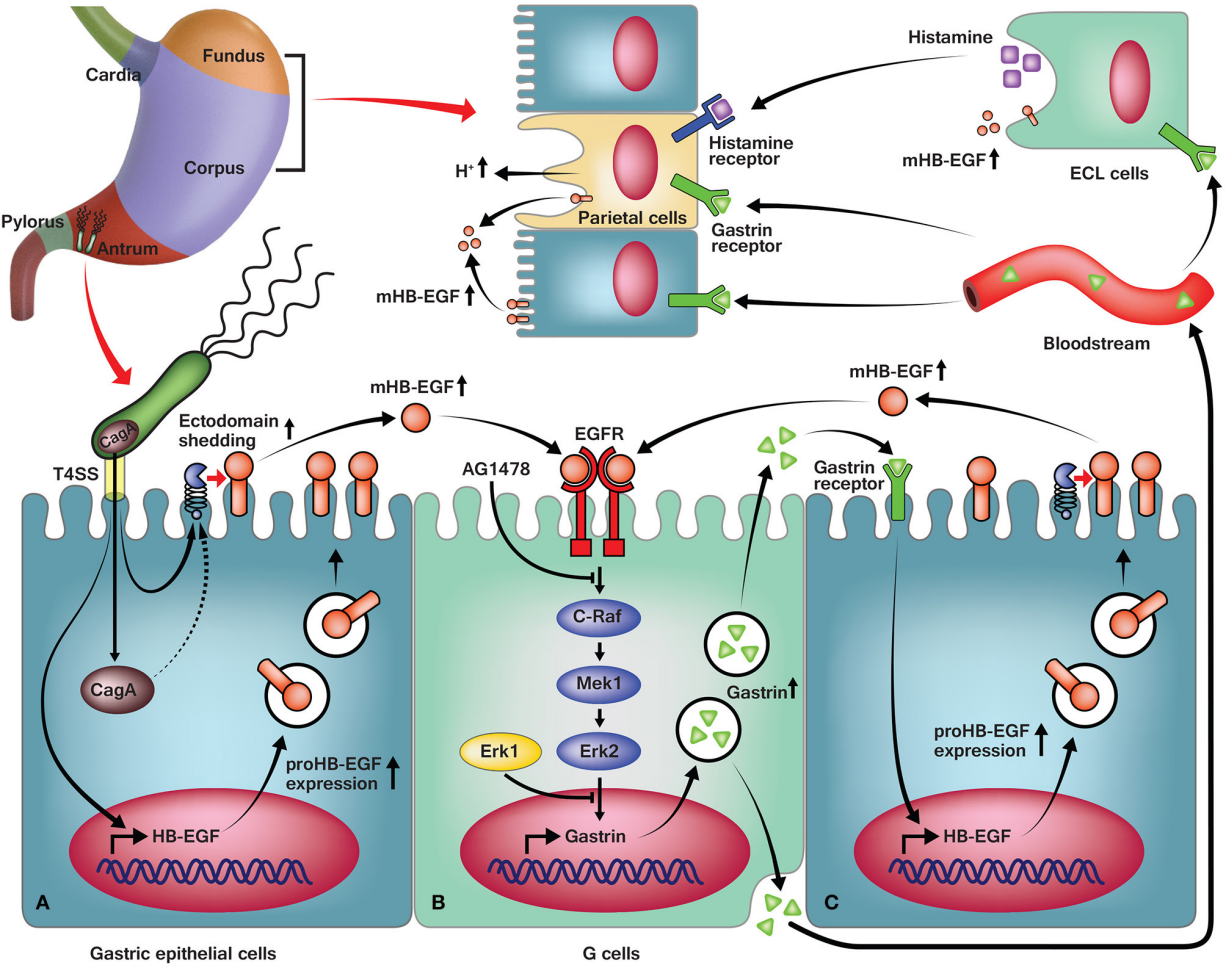
A predictive model of *H. pylori*-induced gastrin expression. **(A)** In antral gastric epithelial cells, *H. pylori* induces expression of HB-EGF via the T4SS, and downstream shedding of HB-EGF via the T4SS and partially, CagA. **(B)** Increased mature HB-EGF binds to the EGF receptor in the G cells and activates C-Raf, Mek1, and Erk2 in the Erk pathway, resulting in an increase of gastrin expression. **(C)** The secreted gastrin is transported to its site of action in paracrine and endocrine manners, and upregulates HB-EGF expression in the antrum, corpus, and fundus. (Top) Also, the gastrin stimulates ECL cells to produce more histamine, which co-stimulates parietal cells with gastrin, to induce gastric acid secretion.

*En masse*, the data presented herein leads to a detailed molecular model of *H. pylori*-mediated regulation (Figure 7) that can be broken into several distinct phases. The first step in the model involves interaction of *H. pylori* with the gastric epithelial cells (Figure 7A). Consistent with prior results (Rieder et al., 2005; Wiedemann et al., 2012), our data indicate the importance of the T4SS in *H. pylori*-induced gastrin promoter activity. One important function of the T4SS is to inject CagA into host gastric cells; thus, it is possible that CagA translocation may be crucial for gastrin induction. Indeed, the involvement of CagA in the induction of gastrin promoter activity has remained controversial (Tucker et al., 2010; Zhou et al., 2011; Wiedemann et al., 2012). However, our data show that G27Δ*cagA* and K74Δ*cagA* were both able to induce gastrin promoter activity to levels similar to those induced by the WT strain. Conversely, G27ΔPAI failed to induce the gastrin promoter to comparable levels. Additionally, consistent with a previous study (Wiedemann et al., 2012), G27Δ*cagL* and K74Δ*cagL* failed to induce promoter activity; deletion of *cagL* impairs assembly of the T4SS apparatus in *H. pylori*. Therefore, our data strongly suggest that the T4SS apparatus, but not the effector CagA, is essential for *H. pylori*-mediated gastrin induction.

Infection of the gastric epithelial cells with *H. pylori* increased HB-EGF mRNA expression at 1 h and subsequently induced gastrin mRNA expression at 4 h. Intriguingly, the expression change of HB-EGF as well as gastrin was dependent on the T4SS apparatus but independent of CagA (Figure 7A). Moreover, *H. pylori* stimulated HB-EGF ectodomain shedding, which we showed to be an important process for induction of gastrin expression. Our data indicate that mature HB-EGF activates the gastrin promoter via transactivation of the EGF receptor. However, it should be noted that other factors may contribute to the increased activation of EGF receptor; these include increased expression of the EGF receptor (Supplementary Figure 3B) and other EGF receptor ligands (Figure 2). Hitherto, EGF was the only EGF growth factor family member known to induce gastrin expression (Godley and Brand, 1989; Ford et al., 1997; Chupreta et al., 2000). Despite the fact that *H. pylori* infection induced EGF mRNA expression, it was surprising that the knockdown of EGF by siRNA did not reduce *H. pylori*-induced gastrin promoter activity. As shown in Figure 2, the absolute quantification of mRNA copy number showed that *H. pylori*-induced expression of EGF mRNA was minor in comparison to HB-EGF mRNA expression. Additionally, EGF protein expression was not able to be detected in AGS cells pre- or post *H. pylori* infection via Western blot analysis (data not shown). Because we found that the recombinant EGF protein could induce gastrin promoter activation (Figure 4A), we conclude that the lack of effect of EGF siRNA treatment on *H. pylori*-induced gastrin expression is probably due to the low level of EGF expression. In addition, it could be possible that the lack of effect of AR knockdown on gastrin expression would be overshadowed by HB-EGF induction upon AR siRNA treatment (Figure 3F). However, rAR treatment did not induce gastrin promoter activation (Figure 4A) and thus, AR expression is not required for gastrin induction by *H. pylori* infection.

Thus, our data indicate that HB-EGF is a previously unrecognized EGF family member that functions as a gastrin promoter activator (Figure 7B). Interestingly, several groups have reported that gastrin acts on the CCK-2 receptor to induce HB-EGF expression and shedding (Figure 7C) (Dickson et al., 2006; Yin et al., 2010). However, the involvement of HB-EGF in *H. pylori*-induced gastrin expression seen in our study must be an earlier upstream event since siRNA knockdown of HB-EGF resulted in a complete loss of *H. pylori*-induced gastrin expression. Taken *en masse, H. pylori* may control a double positive feedback loop between HB-EGF and gastrin expression (Figure 7).

MAPK pathways have been extensively studied; hence several studies have focused on involvement of the Erk pathway in gastrin expression (Merchant et al., 1999; Chupreta et al., 2000). For our work, an EGF receptor kinase inhibitor or specific siRNAs targeting each isoforms of Raf, Mek and Erk were used to define how the Erk pathway is involved in *H. pylori*-induced gastrin expression. We made several novel and interesting findings. Our data indicate significant roles for the EGF receptor, C-Raf, Mek1, and Erk2 in *H. pylori*-mediated gastrin promoter activity (Figure 7B). Conversely, siRNA knockdown of A-Raf, B-Raf or Mek2 did not alter gastrin promoter activity. Previous *in vitro* experiments using several gastric cancer cell lines showed that oncogenic H- and K-Ras activate the gastrin promoter, thus suggesting the importance of various Ras isoforms on gastrin expression (Nakata et al., 1998). That previous finding is consistent with our results since it is known that the H-Ras isoform mainly activates C-Raf. One important aspect of Erk cascade regulation revealed by our data is the specific, non-redundant roles of protein isoforms in this pathway. Indeed, our data indicate that Mek1, but not Mek2, plays an essential role in *H. pylori*-stimulated gastrin promoter activation. Although the Mek1 and Mek2 isoforms are highly homologous (80% similar in overall sequence and 90% similar in their kinase domains; Roskoski, 2012), a role for Mek1 but not Mek2 in *H. pylori*-stimulated gastrin promoter activity further supports the non-redundant functions of the Mek isoforms (Alessandrini et al., 1997; Skarpen et al., 2008; Scholl et al., 2009; Zhou et al., 2010). Furthermore, siRNA knockdown of Erk1 and Erk2 revealed unexpected and intriguing results; knockdown of Erk1 induced gastrin promoter activity, while knockdown of Erk2 resulted in a significant reduction in the promoter activity. These data suggest that in the context of gastrin expression, Erk1 acts as a repressor, whereas Erk2 acts as a potent activator. Recent results from *in vivo* as well as *in vitro* experiments (Pagès et al., 1999; Yao et al., 2003; Vantaggiato et al., 2006) likewise provide convincing evidence that the Erk isoforms have distinct functions; this is despite a sequence identity of 84% between Erk1 and Erk2 and their equal participation in many cellular functions (Roskoski, 2012). One possible hypothesis for the opposite roles of these isoforms, is that since the Erk proteins are the only known Mek kinases substrates, perhaps Erk1 and Erk2 compete as Mek kinase substrates (Girault et al., 2007). Lack of Erk1 could thus, induce higher levels of Erk2 phosphorylation. However, western blot analysis of Erk2 levels during knockdown of Erk1 in G27 or PMSS1 did not show an obvious difference at the level of Erk2 phosphorylation. Indeed, gastrin promoter activation levels were not correlated with the pattern of Erk2 phosphorylation. Another possibility is that Erk1 and Erk2 activate different transcription factors, which then have different activities (repressing and activating, respectively) on the downstream promoters. It has previously been shown that Erk2 activation of the Sp1 transcription factor is important for EGF-induced-gastrin promoter activation (Chupreta et al., 2000). Finally, Erk1 and Erk2 may function as homodimers and heterodimers in the cytoplasm (Casar et al., 2008) and thus, the Erk2 homodimer may act as a more potent activator than the Erk1 and 2 heterodimer in the induction of gastrin expression. Clearly, further studies are necessary to understand the distinct roles of the Erk isoforms in *H. pylori*-stimulated gastrin expression. It is worth noting that, though too complicated to include in our model, other stress signals may also cause activation of MAPKs that then effect *H. pylori*-induced gastrin expression.

Taken *en masse*, our data demonstrate that *H. pylori* strains carrying a functional T4SS apparatus induce gastrin promoter activity *via* HB-EGF, the EGF receptor, C-Raf, Mek1, and Erk2 in the Erk pathway. A greater understanding of the involvement of the HB-EGF-mediated EGF receptor transactivation and the MAPK pathway, especially the negative role played by Erk1, in *H. pylori*-induced gastrin expression may help in the development of therapeutic strategies to reduce *H. pylori*-induced hypergastrinemia and consequent development of gastric cancers.

## Author contributions

NG, DM, JK, and J-HC: Designed the study. NG, SJ, YC, YH, and JK: Performed the experiment. NG, SJ, YC, Y-EJ, AK, HS, J-HK, Y-JY, and JK: Analyzed the data. NG, SJ, JK, and J-HC: Drafted the manuscript. SJ, DM, J-HC: Revised the manuscript.

### Conflict of interest statement

The authors declare that the research was conducted in the absence of any commercial or financial relationships that could be construed as a potential conflict of interest.

## Supplementary material

Supplementary Table 1 Sequences of siRNAs used in this study.

Supplementary Table 2 Specific primers utilized for qRT-PCR.

Supplementary Figure 1 *H. pylori* infection increases gastrin expression in gastric epithelial cells. Gastrin mRNA expression was measured by qRT-PCR using RNA isolated at the indicated times after AGS cells were infected with *H. pylori* G27 WT. ^*^*P* < 0.05 compared to 0 h.

Supplementary Figure 2 Field emission scanning electron microscopy analysis of the T4SS apparatus of *H. pylori* co-cultured with AGS cells. AGS cells were co-cultured at a MOI of 100 for 4 h with wild-type *H. pylori* strains G27 or K74, as well as their isogenic mutant derivatives, Δ*cagA* and Δ*cagL*, to analyze the presence of T4SS by high resolution field emission scanning electron microscopy. Arrows indicate the T4SS apparatus.

Supplementary Figure 3 Effect of *H. pylori* infection on EGFR-mediated MAPK activation. **(A)** Erk activation was measured by Western blot at the indicated time points after AGS cells were infected with *H. pylori* G27 WT for the indicated durations, with or without pretreatment of AG1478 (1 μM) for 30 min. **(B)** AGS cells were infected with G27 WT, G27Δ*cagA*, and G27ΔPAI for 5 h, and activation and expression of EGFR were measured by Western blot. GAPDH and Urease A were used as loading controls for AGS cell and *H. pylori*, respectively.

Supplementary Figure 4 siRNA Knockdown of HB-EGF does not alter induction of IL-8 secretion. AGS cells were treated with NT siRNA (100 pM), or HB-EGF siRNA (50 and 100 pM). At 48 h after siRNA treatment, cells were infected with *H. pylori* strain G27 at an MOI of 100 for 5 h. Bar graphs indicate mean IL-8 secretion and error bars indicate standard deviation. ^**^*P* < 0.01.

Supplementary Figure 5 siRNA Knockdown of HB-EGF does not alter cell elongation. AGS cells were treated with NT siRNA (100 pM), or HB-EGF siRNA (50 and 100 pM). At 48 h after siRNA treatment, cells were infected with *H. pylori* strain G27 at an MOI of 100 for 5 h. Cells were fixed with 4% paraformaldehyde and images were taken under ×200 magnification.
