# Supplementary material for: Helicobacter pylori-Induced HB-EGF Upregulates Gastrin Expression via the EGF Receptor, C-Raf, Mek1, and Erk2 in the MAPK Pathway
Source: Front Cell Infect Microbiol. 2018 Jan 15;7:541. doi: 10.3389/fcimb.2017.00541 (PMC5775237; doi:10.3389/fcimb.2017.00541)
Supplement: Supplementary Table 1 — Sequences of siRNAs used in this study. [file Table1.docx]

***Supplementary Material***

***Helicobacter pylori*-induced HB-EGF Upregulates Gastrin Expression via the EGF Receptor, C-Raf, Mek1, and Erk2 in the MAPK Pathway**

**Niluka Gunawardhana^1, 2¶^, Sungil Jang^1¶^, Yun Hui Choi^1^, Youngmin A. Hong^1^, Yeong-Eui Jeon^1^, Aeryun Kim^1^, Hanfu Su^1, 3^, Ji-Hye Kim^4^, Yun-Jung Yoo^1^, D. Scott Merrell^5*^, Jinmoon Kim^1*#^, and Jeong-Heon Cha^1, 3*^**

^1^ Department of Oral Biology, Oral Science Research Center, Department of Applied Life Science, The Graduate School, BK21 Plus Project, Yonsei University College of Dentistry, Seoul, Republic of Korea

^2^ Department of Basic Sciences, Faculty of Dental Sciences, University of Peradeniya, Peradeniya, Sri Lanka

^3^ Microbiology & Molecular Biology, Key Laboratory of Oral Medicine, Guangzhou Institute of Oral Disease, Stomatology Hospital of Guangzhou Medical University, Guangzhou, China

^4^ Department of Dental Hygiene, Jeonju Kijeon College, Jeonju, Republic of Korea

^5^ Department of Microbiology and Immunology, Uniformed Services University of the Health Sciences, Bethesda, Maryland, USA

^#^ Current Address: ATGen Ltd., Seongnam-si, Gyeonggi-do, Republic of Korea

^¶^ These authors contributed equally to this work.

*** Correspondence:**

Jeong-Heon Cha: jcha@yuhs.ac

Jinmoon Kim: jmkim@nkmax.co.kr

D. Scott Merrell: douglas.merrell@usuhs.edu

**Supplementary Table 1. Sequences of siRNAs used in this study**

| Target | Sequences (5′ → 3′) | References |
| --- | --- | --- |
| NT | AAA CCG UCG AUU UCA CCC GGG | (Martin et al., 2012) |
| HB-EGF | 1. ATC GCT TAT ATA CCT ATG A  2. AGG AGG TTA TGA TGT GGA A | This study  This study |
| AR | 1. CGA ACC ACA AAU ACC UGG C  2. CCU GGA AGC AGU AAC AUG C | (Busser et al., 2010)  (Busser et al., 2010) |
| EGF | 1. AAU CCU UAU GAG GAG UCG A  2. CCA CCA CUA UUC CGU AAG A | (Casalino-Matsuda et al., 2004)  TRCN0000055479, RNAi Consortium human shRNA library (Broad Institute, MIT) |
| TGF-α | 1. AAU GAC UGC CCA GAU UCC CAC  2. GUG GGA AUC UGG GCA GUC AUU | This study  This study |
| A-Raf | 1. CGA GAU CUC AAG UCU AAC A  2. GCU UCC AGU CAG ACG UCU A  3. GGA CUC CUC UCU UUC UUC A | Santa Cruz Biotechnology, Catalog No. sc-29615  Santa Cruz Biotechnology, Catalog No. sc-29615  Santa Cruz Biotechnology, Catalog No. sc-29615 |
| B-Raf | AAA GAA UUG GAU CUG GAU CAU | (Pardo et al., 2006) |
| C-Raf | UAG UUC AGC AGU UUG GCU A | (Sheridan et al., 2008) |
| Mek1 | GCU UCU AUG GUG CGU UCU ACA | (Johannessen et al., 2010) |
| Mek2 | UGG ACU AUA UUG UGA ACG AGC | (Johannessen et al., 2010) |
| Erk1 | AAC UUG UAC AGG UCA GUC U | (Kolb et al., 2012) |
| Erk2 | AAU AAG UCC AGA GCU UUG G | (Kolb et al., 2012) |

**Supplementary Table 2. Specific primers utilized for qRT-PCR**

| Gene | Primer sequences (5′ → 3′) | Annealing temp. (°C) | Product size (bp) | References |
| --- | --- | --- | --- | --- |
| Gastrin | F: CCC AGG CTC TCA TCA TCG AAG G  R: GCC GAA GTC CAT CCA TCC ATA GG | 60 | 127 | (Tucker et al., 2010) |
| HB-EGF | F: GTT CTC TCG GCA CTG GTG A  R: TGG TCC GTG GAT ACA GTG G | 55 | 101 | This study |
| AR | F: CGG GAG CCG ACT ATG ACT AC  R: CCA TTT TTG CCT CCC TTT TT | 62 | 172 | (Morita et al., 2007) |
| EGF | F: GCA GAT GGG TCA ATG CAA C  R: GGG ACA GGA GCC CTT ATC A | 62 | 111 | This study |
| TGF-α | F: GCA GAT GGG TCA ATG CAA C  R: GGG ACA GGA GCC CTT ATC A | 62 | 90 | This study |
| 18s rRNA | F: CGG CTA CCA CAT CCA AGG AA  R: GCT GGA ATT ACC GCG GCT | 56 | 187 | (Luther et al., 2005) |

**Supplementary references**

Busser, B., Sancey, L., Josserand, V., Niang, C., Favrot, M.C., Coll, J.L., et al. (2010). Amphiregulin promotes BAX inhibition and resistance to gefitinib in non-small-cell lung cancers. *Mol Ther* 18(3)**,** 528-535. doi: 10.1038/mt.2009.226.

Casalino-Matsuda, S.M., Monzon, M.E., Conner, G.E., Salathe, M., and Forteza, R.M. (2004). Role of hyaluronan and reactive oxygen species in tissue kallikrein-mediated epidermal growth factor receptor activation in human airways. *J Biol Chem* 279(20)**,** 21606-21616. doi: 10.1074/jbc.M309950200.

Johannessen, C.M., Boehm, J.S., Kim, S.Y., Thomas, S.R., Wardwell, L., Johnson, L.A., et al. (2010). COT drives resistance to RAF inhibition through MAP kinase pathway reactivation. *Nature* 468(7326)**,** 968-972. doi: 10.1038/nature09627.

Kolb, R.H., Greer, P.M., Cao, P.T., Cowan, K.H., and Yan, Y. (2012). ERK1/2 signaling plays an important role in topoisomerase II poison-induced G2/M checkpoint activation. *PLoS One* 7(11)**,** e50281. doi: 10.1371/journal.pone.0050281.

Luther, C., Wienhold, W., Oehlmann, R., Heinemann, M.K., Melms, A., and Tolosa, E. (2005). Alternatively spliced transcripts of the thymus-specific protease PRSS16 are differentially expressed in human thymus. *Genes Immun* 6(1)**,** 1-7. doi: 10.1038/sj.gene.6364142.

Martin, M.J., Hayward, R., Viros, A., and Marais, R. (2012). Metformin accelerates the growth of BRAF V600E-driven melanoma by upregulating VEGF-A. *Cancer Discov* 2(4)**,** 344-355. doi: 10.1158/2159-8290.CD-11-0280.

Morita, S., Shirakata, Y., Shiraishi, A., Kadota, Y., Hashimoto, K., Higashiyama, S., et al. (2007). Human corneal epithelial cell proliferation by epiregulin and its cross-induction by other EGF family members. *Mol Vis* 13**,** 2119-2128.

Pardo, O.E., Wellbrock, C., Khanzada, U.K., Aubert, M., Arozarena, I., Davidson, S., et al. (2006). FGF-2 protects small cell lung cancer cells from apoptosis through a complex involving PKCepsilon, B-Raf and S6K2. *EMBO J* 25(13)**,** 3078-3088. doi: 10.1038/sj.emboj.7601198.

Sheridan, C., Brumatti, G., and Martin, S.J. (2008). Oncogenic B-RafV600E inhibits apoptosis and promotes ERK-dependent inactivation of Bad and Bim. *J Biol Chem* 283(32)**,** 22128-22135. doi: 10.1074/jbc.M800271200.

Tucker, T.P., Gray, B.M., Eaton, K.A., and Merchant, J.L. (2010). Helicobacter pylori induction of the gastrin promoter through GC-rich DNA elements. *Helicobacter* 15(5)**,** 438-448. doi: 10.1111/j.1523-5378.2010.00787.x.
