# Supplementary figures and images for: Helicobacter pylori-Induced HB-EGF Upregulates Gastrin Expression via the EGF Receptor, C-Raf, Mek1, and Erk2 in the MAPK Pathway
Source: Front Cell Infect Microbiol. 2018 Jan 15;7:541. doi: 10.3389/fcimb.2017.00541 (PMC5775237; doi:10.3389/fcimb.2017.00541)

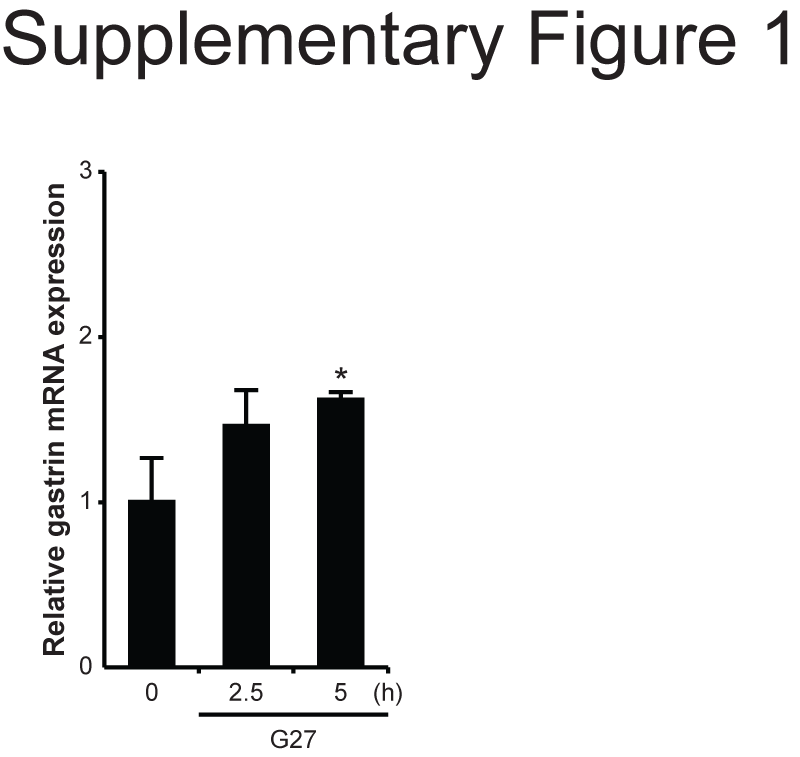

Supplement: Supplementary Figure 1 — H. pylori infection increases gastrin expression in gastric epithelial cells. Gastrin mRNA expression was measured by qRT-PCR using RNA isolated at the indicated times after AGS cells were infected with H. pylori G27 WT. *P < 0.05 compared to 0 h. [file Image1.TIF]

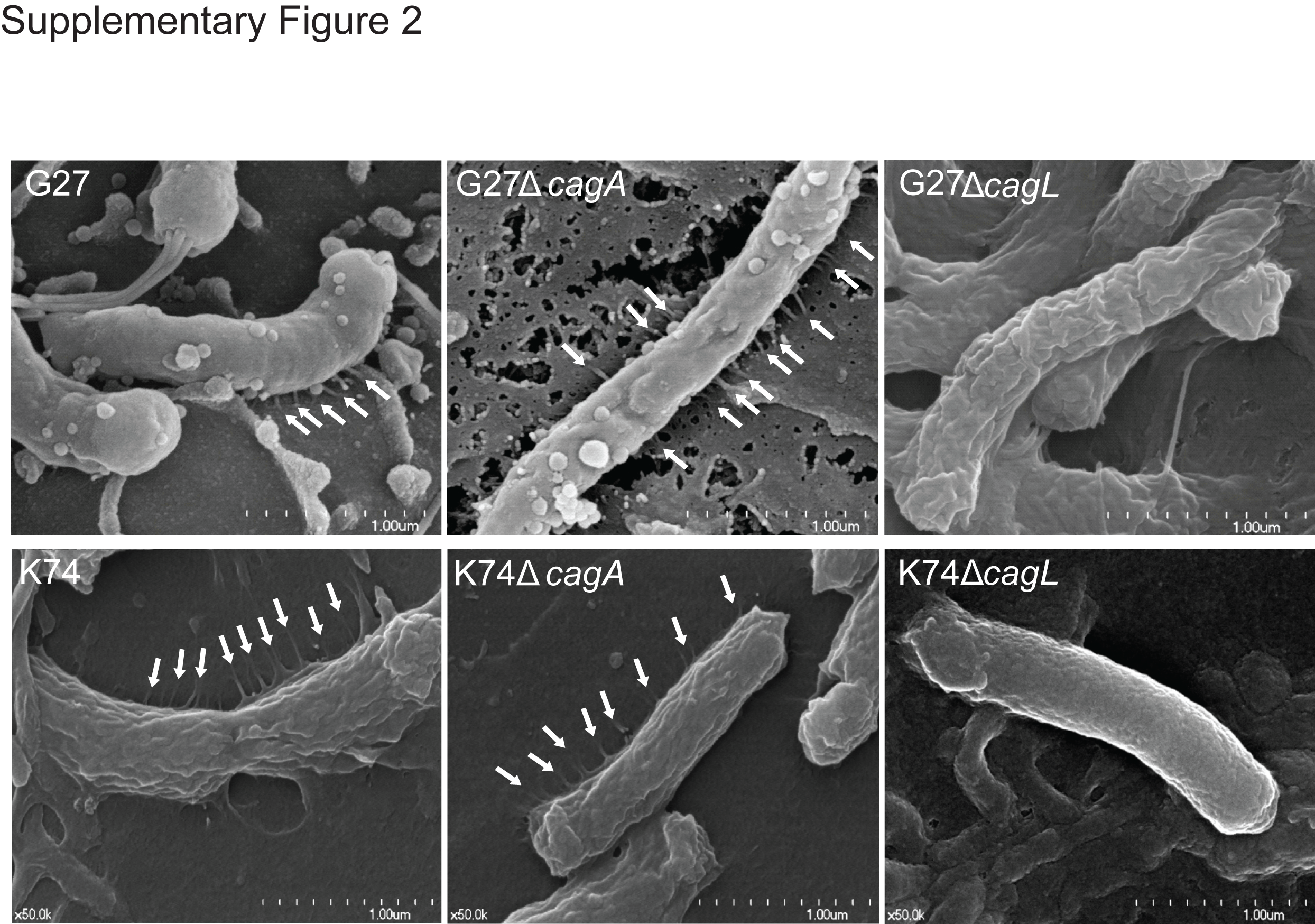

Supplement: Supplementary Figure 2 — Field emission scanning electron microscopy analysis of the T4SS apparatus of H. pylori co-cultured with AGS cells. AGS cells were co-cultured at a MOI of 100 for 4 h with wild-type H. pylori strains G27 or K74, as well as their isogenic mutant derivatives, ΔcagA and ΔcagL, to analyze the presence of T4SS by high resolution field emission scanning electron microscopy. Arrows indicate the T4SS apparatus. [file Image2.TIF]

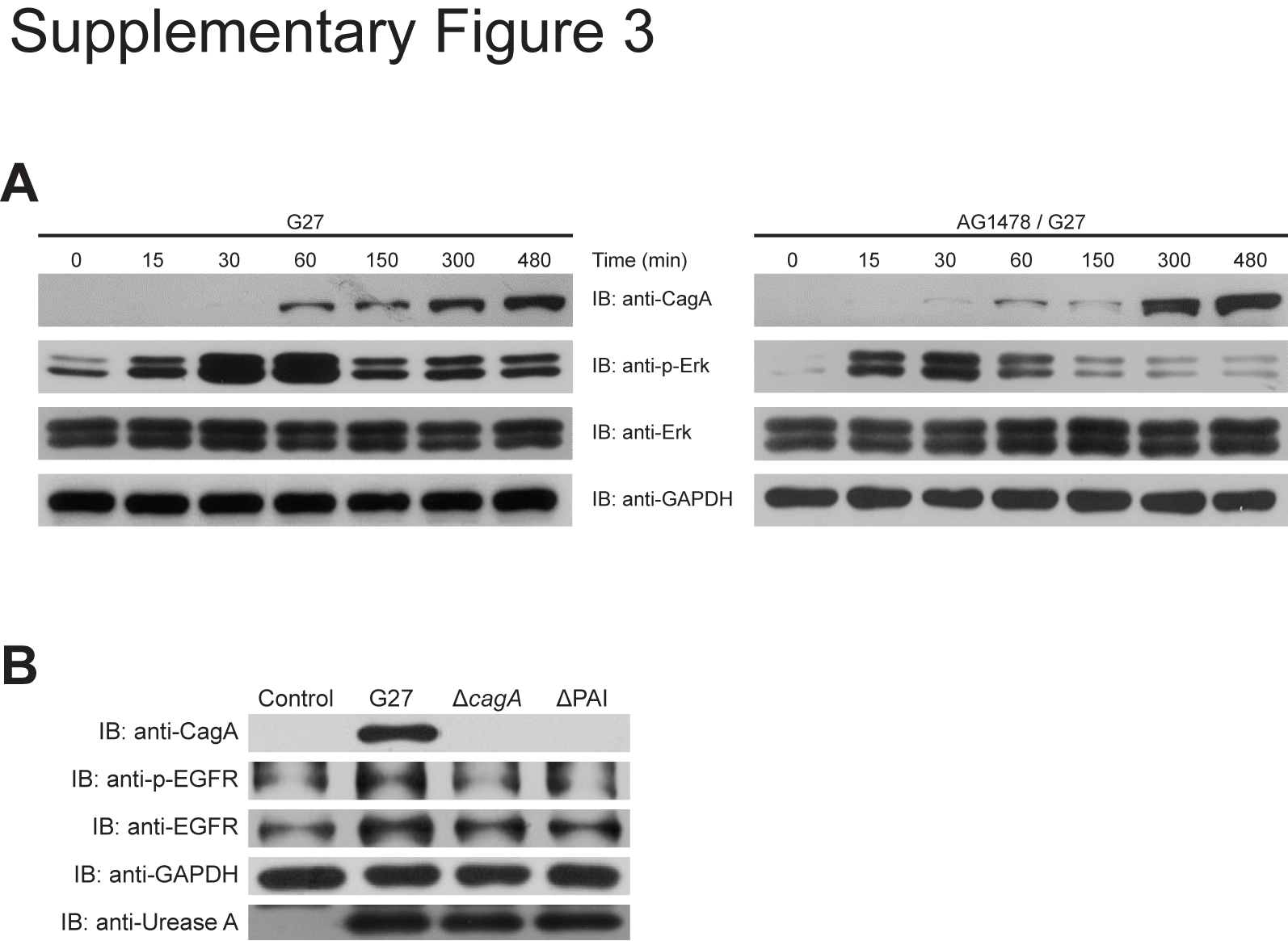

Supplement: Supplementary Figure 3 — Effect of H. pylori infection on EGFR-mediated MAPK activation. (A) Erk activation was measured by Western blot at the indicated time points after AGS cells were infected with H. pylori G27 WT for the indicated durations, with or without pretreatment of AG1478 (1 μM) for 30 min. (B) AGS cells were infected with G27 WT, G27ΔcagA, and G27ΔPAI for 5 h, and activation and expression of EGFR were measured by Western blot. GAPDH and Urease A were used as loading controls for AGS cell and H. pylori, respectively. [file Image3.TIF]

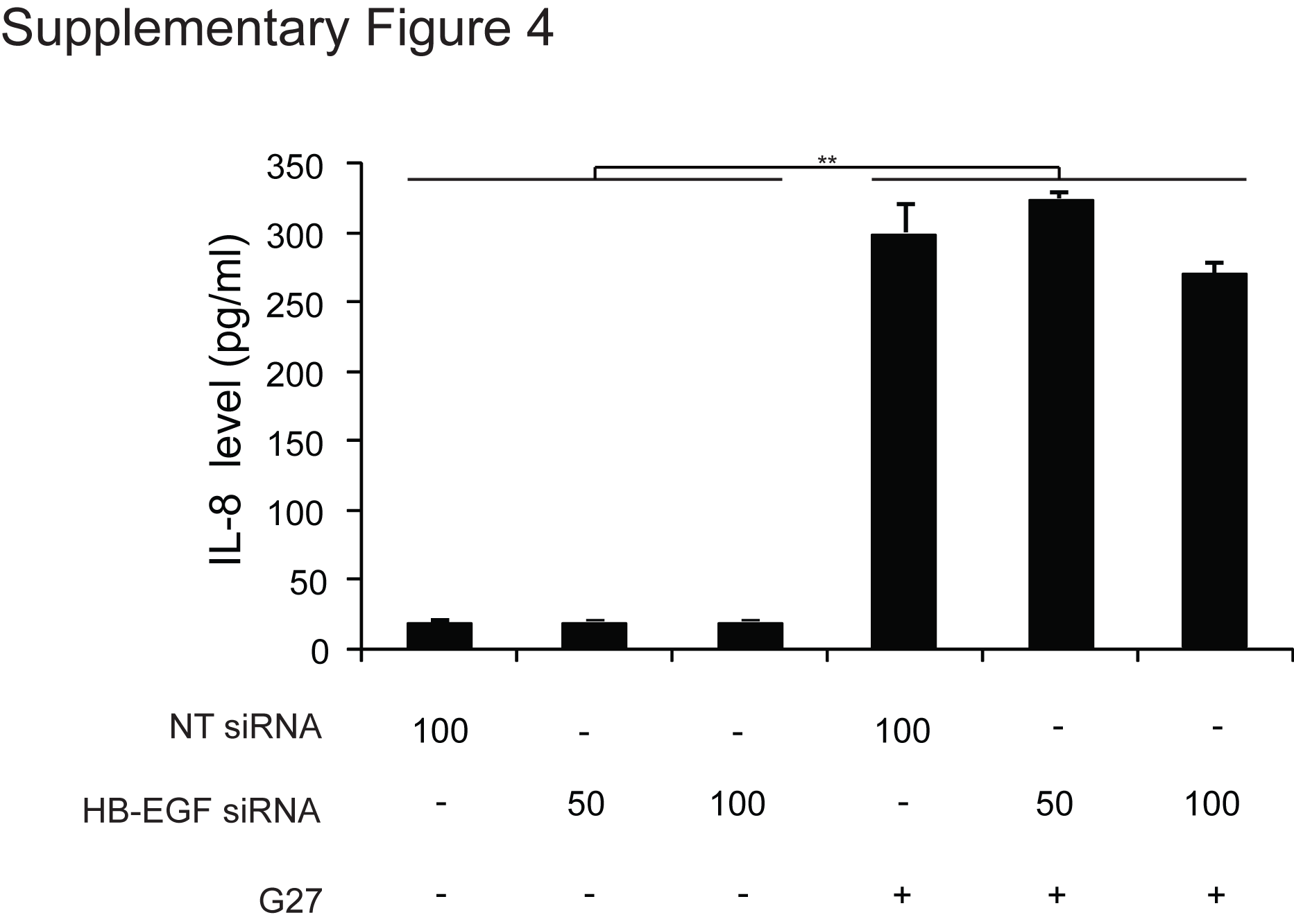

Supplement: Supplementary Figure 4 — siRNA Knockdown of HB-EGF does not alter induction of IL-8 secretion. AGS cells were treated with NT siRNA (100 pM), or HB-EGF siRNA (50 and 100 pM). At 48 h after siRNA treatment, cells were infected with H. pylori strain G27 at an MOI of 100 for 5 h. Bar graphs indicate mean IL-8 secretion and error bars indicate standard deviation. **P < 0.01. [file Image4.TIF]

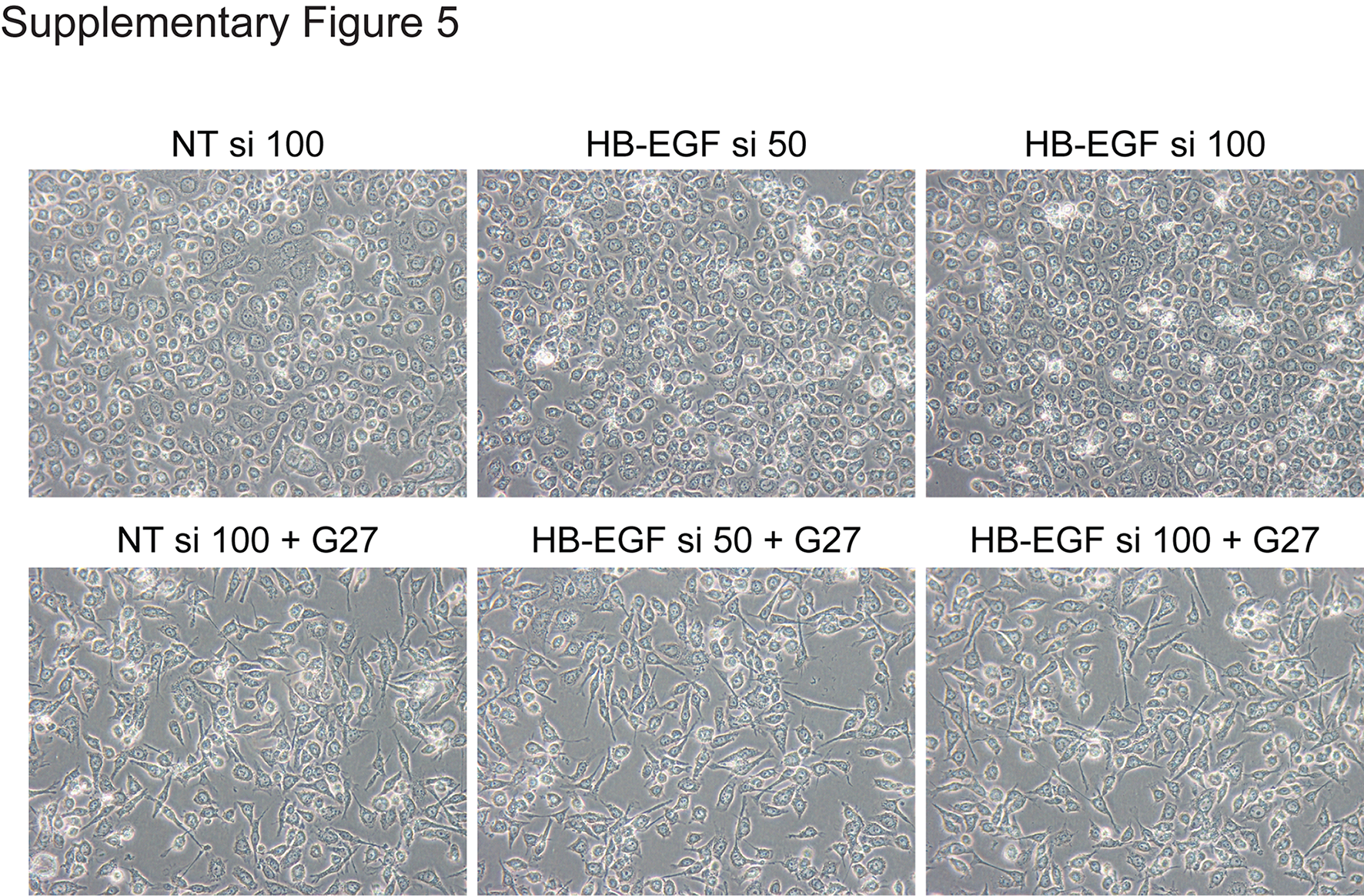

Supplement: Supplementary Figure 5 — siRNA Knockdown of HB-EGF does not alter cell elongation. AGS cells were treated with NT siRNA (100 pM), or HB-EGF siRNA (50 and 100 pM). At 48 h after siRNA treatment, cells were infected with H. pylori strain G27 at an MOI of 100 for 5 h. Cells were fixed with 4% paraformaldehyde and images were taken under ×200 magnification. [file Image5.TIF]
